# Nematode chromosomes

**DOI:** 10.1093/genetics/iyac014

**Published:** 2022-03-22

**Authors:** Peter M Carlton, Richard E Davis, Shawn Ahmed

**Affiliations:** 1 Graduate School of Biostudies, Kyoto University, Kyoto 606-8501, Japan; 2 Department of Biochemistry and Molecular Genetics, University of Colorado School of Medicine, Denver, CO 80045, USA; 3 RNA Bioscience Initiative, University of Colorado School of Medicine, Aurora, CO 80045, USA; 4 Department of Genetics, University of North Carolina, Chapel Hill, NC 27599, USA; 5 Department of Biology, University of North Carolina, Chapel Hill, NC 27599, USA

**Keywords:** holocentric, synteny, centromere, telomere, programmed DNA elimination, meiosis, repetitive DNA, WormBook

## Abstract

The nematode *Caenorhabditis elegans* has shed light on many aspects of eukaryotic biology, including genetics, development, cell biology, and genomics. A major factor in the success of *C. elegan*s as a model organism has been the availability, since the late 1990s, of an essentially gap-free and well-annotated nuclear genome sequence, divided among 6 chromosomes. In this review, we discuss the structure, function, and biology of *C. elegans* chromosomes and then provide a general perspective on chromosome biology in other diverse nematode species. We highlight malleable chromosome features including centromeres, telomeres, and repetitive elements, as well as the remarkable process of programmed DNA elimination (historically described as chromatin diminution) that induces loss of portions of the genome in somatic cells of a handful of nematode species. An exciting future prospect is that nematode species may enable experimental approaches to study chromosome features and to test models of chromosome evolution. In the long term, fundamental insights regarding how speciation is integrated with chromosome biology may be revealed.

## Introduction 

Chromosome biology has a noted history in nematodes, beginning with studies in the late 19th century on a parasitic nematode of horses (originally called *Ascaris megalocephala*, but now known as *Parascaris univalens* or *Parascaris equorum*). Edouard van Beneden identified *Parascaris* as an organism with attributes that made it an exceptional model at that time for the study of chromosomes. These attributes included the ability to obtain and observe stages of gametogenesis in the long reproductive organs (male and female gonads with lengths on average of 70 cm or more), large sperm, large numbers of oocytes and clear eggs, synchronous and slow early development, and the presence of very large chromosomes (genome size of ∼2.5 Gb in *P. univalens* with 2*N* = 2). Van Beneden (1883) used *Parascaris* to observe and describe the processes of gametogenesis, including the reductive divisions of meiosis, and re-establishment of the diploid number following fusion of the egg and sperm. He also described the constancy of chromosomes in cells and within a species. Theodor Boveri capitalized on this emerging cell biology model, using it to help develop the theory of chromosome inheritance, the chromosome cycle, and the key role of centrosomes in chromosome segregation. The chromosomal basis of sex determination was also proposed by Boveri. These were fundamental observations that changed our view of chromosomes and inheritance and foreshadowed many findings to come. The works of van Beneden and Boveri have been described and their importance discussed in a number of excellent reviews (Baltzer 1964; Hamoir 1992; Moritz and Sauer 1996; Sathananthan *et al.* 2006; Maderspacher 2008; Satzinger 2008; Dietel 2014). 

Our understanding of chromosomes and their organization and function have been greatly enhanced by the development of *Caenorhabditis elegans* as a major model organism over the past 50 years. In this review, we focus on the chromosomes of *C. elegans* and its nematode relatives, including some distantly related parasitic and free-living species. We discuss chromosome size and complexity, genomes, sex chromosomes, dispersed centromeres that are holocentric rather than monocentric, and programmed DNA elimination. We endeavor to provide insights into common and divergent properties of nematode chromosomes and their genome sequences.

## Section 1: Chromosome biology of *C. elegans*

### Genome size and chromosomal organization

**Fig. 1.**
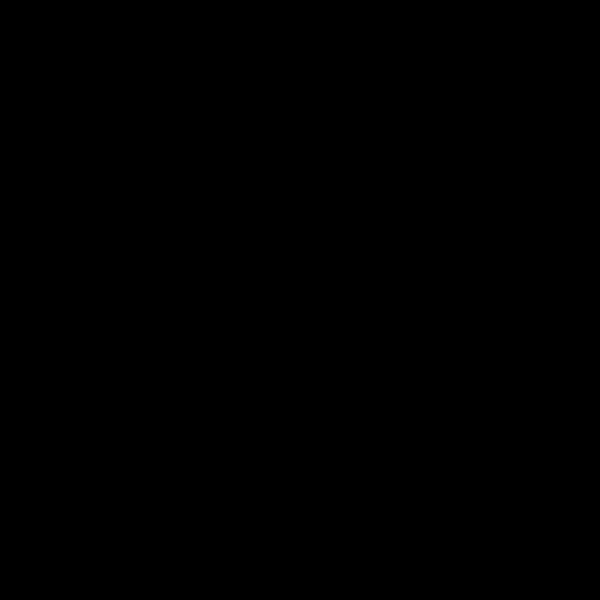
The 6 chromosomes of *C. elegans* at the pachytene stage of meiosis, after pairing and synapsis, visualized with 3D-SIM microscopy. Chromosomes have been pseudocolored after manual tracing and segmentation.

The complement of chromosomes defines an organism’s nuclear genome. The first wholly sequenced genome from a multicellular organism was that of the Bristol N2 strain of the nematode *C. elegans*, which has been a central focus of many experimental biologists. Bristol N2 genomic DNA isolated in the 1990s was cloned into a series of BACs and YACs whose sequences were used to create contiguous segments of genome sequence (contigs; Coulson *et al.* 1995). This physical map was integrated with and anchored to a detailed genetic map derived from recombination frequencies between many genes that had been identified by mutation on 6 linkage groups. The remaining gaps in the genome were manually filled in using finishing approaches that crossed gaps or isolated telomeres. These painstaking efforts created a 100.3 Mb genome assembly that was essentially free of gaps, with 6 contigs corresponding to the 6 *C. elegans* chromosomes (Fig. 1) and a seventh small contig that corresponds to the mitochondrial genome (The *C. elegans* Sequencing Consortium 1998; Hillier *et al.* 2005). This essentially complete genome assembly has experienced minor contemporary improvements to correct artifacts (Tyson *et al.* 2018; Yoshimura *et al.* 2019).

The general organization of *C. elegans* autosomes was revealed by the initial genome assembly (The *C. elegans* Sequencing Consortium 1998). The middle third of each autosome is enriched for conserved genes (based on homology with yeast genes) and contains fewer repetitive sequences compared to the distal thirds (the chromosome “arms”), which are repeat-rich and have fewer essential genes, that in general are not well-conserved. Phenotypic evidence for enrichment of essential genes in the central third of each autosome was provided by genome-wide analysis of gene function based on RNA interference (Kamath *et al.* 2003). Although the *C. elegans* X chromosome has the same arm-center-arm domain structure of autosomes with regard to higher levels of recombination on chromosome arms (Rockman and Kruglyak 2009), it does not possess analogous regions enriched for conserved genes and is visibly and functionally heterochromatic in the germline (Kelly *et al.* 2002). It is possible that the differential requirement for expression in germ cells has led to changes in gene content on the X chromosome, specifically enrichment for somatically expressed genes that control postembryonic phenotypes such as behavior, and depletion of germline genes that are essential for development or fertility (Kamath *et al.* 2003).

**Fig. 2.**
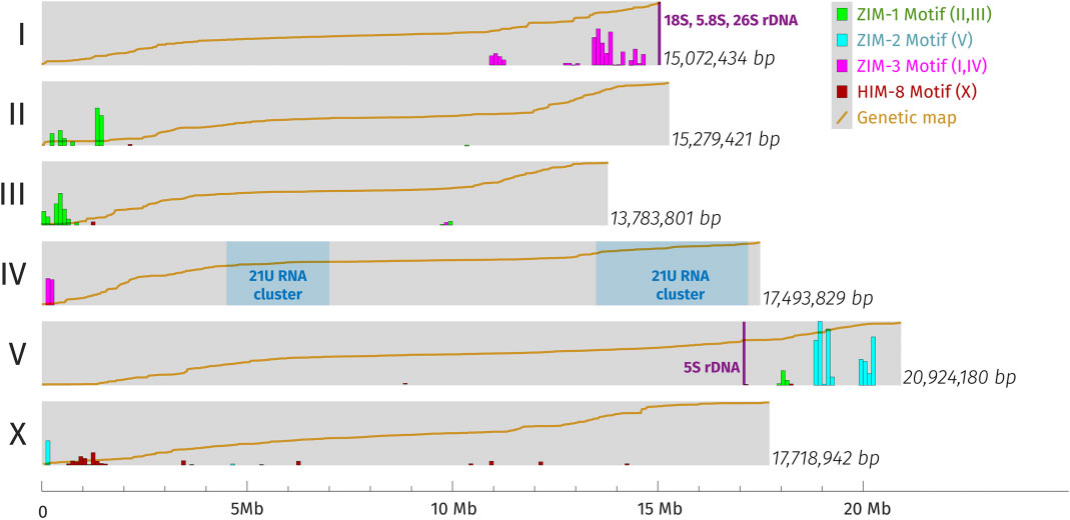
A schematic of the 6 chromosomes of *C. elegans*, based on data collected from Wormbase (Harris *et al.* 2020) version WS280. The chromosomes are shown with their genetically defined left ends at left. The *X*-axis is the physical (genomic) size in megabases. Various landmarks discussed in the text are shown, including large noncoding RNA loci (21U RNAs and ribosomal RNAs) and PC motifs associated with particular ZIM and HIM-8 zinc-finger proteins, shown as density within 100-kb regions (bars). The genetic map position (including both measured and estimated data) of all protein-coding genes in Wormbase is also shown as a yellow line; each line is scaled to fill the height of the horizontal bar and covers ∼50 cM.

Comparison of recombination distances between genes identified by mutations with physical distances based on genome sequencing has revealed differences between genetic map units and DNA base pairs along each chromosome. That variable relationship is shown in Fig. 2, along with several classes of repeats that are enriched on autosome arms (see also interstitial telomere sequences in Fig. 5b). Additional hallmarks of the *C. elegans* genome include 2 clusters of thousands of tiny piRNA genes that generate 21U-RNAs that scan the genome for foreign genetic elements (Bagijn *et al.* 2012), and tandem repeat tracts of rDNA at the right end of Chromosome I and on the right arm of Chromosome V (Fig. 2).

### Genome rearrangements

Genetic balancers, or balancer chromosomes, are a common and convenient way to maintain *C. elegans* strains that carry mutations with lethal or sterile phenotypes that are not possible to maintain when homozygous. To maintain and analyze such mutations, 3 conditions should be fulfilled. First, a functional copy of the gene in question must be provided. Second, the functional balancer chromosome should be linked to a gene conferring an easily scorable dominant phenotype, so heterozygotes (mutant/+) can be picked for maintenance and homozygotes (mutant/mutant) can be identified for analysis. Ideally, the functional copy can be linked to a recessive embryonic lethal mutation, so that animals that are homozygous for the balancer chromosome will not develop. Third, little or no recombination should occur between the lethal allele and the visible marker; in other words, the above linkage should be nearly 100%. This last condition is needed to prevent the confusing creation of strains lacking both the lethal mutation and the marker. All of these conditions are fulfilled by *balancer chromosomes* (Edgley *et al.* 2006), chromosomes that suppress recombination over large genetic distances and carry visible markers that allow for balancer maintenance.

**Fig. 3.**
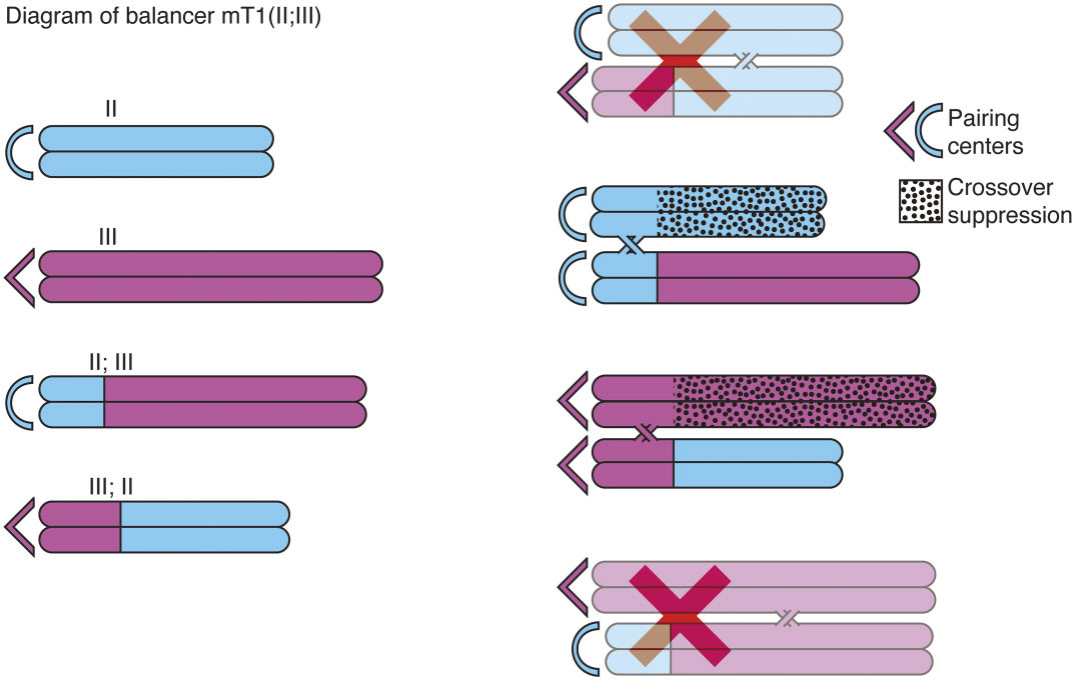
Balancer chromosomes are translocations that suppress crossing over on chromosomes. Light blue: Chromosome II, purple: Chromosome III. Dotted chromosome arms: crossover suppression. Arc/angle symbols at the left end of each chromosome indicate the PC ends which are required in *cis* to initiate synapsis; therefore, the configurations marked with a red X are not observed.

Balancer chromosomes were identified by isolating induced or spontaneous rearrangements of existing chromosomes. Classical balancer chromosomes are of 3 types: duplications, intrachromosomal inversions (including some imperfectly characterized balancers that are most likely inversions), and translocations. Translocations have been the most popular balancers, owing to their extensive genome coverage across segments of distinct chromosomes and robust satisfaction of the above conditions. Due to their utility, the genetic properties of balancer chromosomes are typically very well-characterized. However, since they were constructed through random chromosome breakage and fusion, their structures are not well-understood at the sequence level. Translocations used for balancers can be simple translocations between 2 nonhomologous chromosomes, each with a single breakpoint, where reciprocal swapping of chromosome arms results in 2 new hybrid chromosomes each composed of 2 segments. Detailed observations of the translocation *eT1* revealed that on each hybrid chromosome, crossover recombination occurs normally between one segment and its parental chromosome, but crossovers are suppressed between the other segment and its parental chromosome (Rosenbluth and Baillie 1981); most other translocations behave similarly. These observations eventually led to the elucidation of *C. elegans* pairing centers (PCs), regions near one end of each chromosome that are required in *cis* for pairing of homologous chromosomes and synapsis in meiotic prophase and are present only on the hybrid segments capable of crossing over with their parental chromosomes (see below; Fig. 3).

In addition to translocations, several other chromosome rearrangements can act as balancers. A single fragment of a *C. elegans* chromosome, termed a free duplication, provides wild-type copies of the genes they carry, rarely recombines with full-length chromosomes, and is lost at a high enough frequency to make analysis of recessive phenotypes possible in the context of cell autonomy during development (Hedgecock and Herman 1995; Apfeld and Kenyon 1998). However, loss of duplications is less predictable than loss of translocations, and the genomic regions covered by duplications are rather few, making their use limited. Chromosome inversions suppress recombination by loss of colinearity (since gene order is reversed), but have until recently been limited in their use as well, being rather uncommon. However, Dejima *et al.* (2018) used recent advances in chromosome engineering using CRISPR-Cas9 editing to produce a very useful set of balancer chromosomes that cover 89% of the *C. elegans* genome. Since these balancers involve rearrangement of a single chromosome instead of 2 chromosomes, they do not lead to aneuploidy, and can be used to balance multiple mutations on distinct chromosomes.

### Pairing centers

As stated above, the asymmetric suppression of crossovers in translocation heterozygotes was a key early observation that led to the discovery of PCs, chromosomal regions required in *cis* for homologous chromosome pairing in meiosis. Since translocations are reciprocal between 2 chromosomes, there is no a priori reason to expect crossover suppression to occur at all. In fact, chiasmata can be observed in all segments of heterozygous reciprocal translocations in other species such as maize (Maguire 1989; Maguire and Riess 1994). Therefore, the pattern of asymmetric crossover suppression seen in *C. elegans* translocations suggested that each chromosome has one of its ends specialized to carry out pairing and/or synapsis (McKim *et al.* 1988, 1993; Villeneuve 1994). These ends were termed “homolog recognition regions” and, later, PCs. Limiting homologous pairing and/or synapsis initiation to these sites has the effect of preventing recombination between the translocation breakpoint and the non-PC end (Fig. 3). To determine the sequences underlying the *cis*-acting promotion of pairing, deficiencies of the X chromosome were isolated that led to failure of X segregation, which narrowed down the PC region to the extreme left end of the X (Villeneuve 1994). The protein encoded by the gene *him-8* is specifically necessary for X chromosome disjunction and binds to the PC end of the X chromosome (Phillips *et al.* 2005).

The *him-8* gene is part of an operon of 4 genes that encode closely related C2H2 zinc-finger proteins that bind to 12-base motifs enriched at the terminal chromosome segments genetically defined to be the PC ends (Phillips and Dernburg 2006; Phillips *et al.* 2009). The other 3 proteins are ZIM-1, which binds to the left ends of chromosomes II and III; ZIM-2, which binds the right end of chromosome V; and ZIM-3, which binds the right end of chromosome I and the left end of chromosome IV (see Fig. 2 for positions of these motifs). PC proteins bind to their motifs during early meiotic prophase, where they help tether PC ends of chromosomes to the nuclear envelope, as well as recruit the Polo-like kinase PLK-2 (Harper *et al.* 2011; Labella *et al.* 2011). When thus positioned at the nuclear periphery, chromosome PC ends become attached to a protein complex spanning both inner and outer nuclear envelopes composed of the proteins SUN-1 and ZYG-12, known as the LINC complex (Burke 2018). The LINC complex in turn transduces forces from dynein on cytoskeletal microtubules into movement of chromosomes inside the nucleus (Penkner *et al.* 2009; Sato *et al.* 2009). These PC functions are necessary for timely pairing and synapsis between homologous chromosomes. Experiments with artificially constructed PCs (integrated arrays of PC binding motifs) showed that the use of a particular PC protein is not necessary for specific homologous pairing, since the X chromosome can pair using a ZIM-2-recruiting array in the absence of HIM-8. Similarly, chromosome V can pair using an HIM-8-recruiting array in the absence of ZIM-2 (Phillips *et al.* 2009). These results and the lack of a one-to-one correspondence between PC proteins and chromosomes show that a simple model of homotypic interactions between PC proteins is insufficient to explain homologous pairing specificity. While homotypic interactions between ZIM-bound PCs are found to occur (Phillips *et al.* 2009), their role, if any, in promoting chromosome pairing is not clear.

### Centromeres

**Fig. 4.**
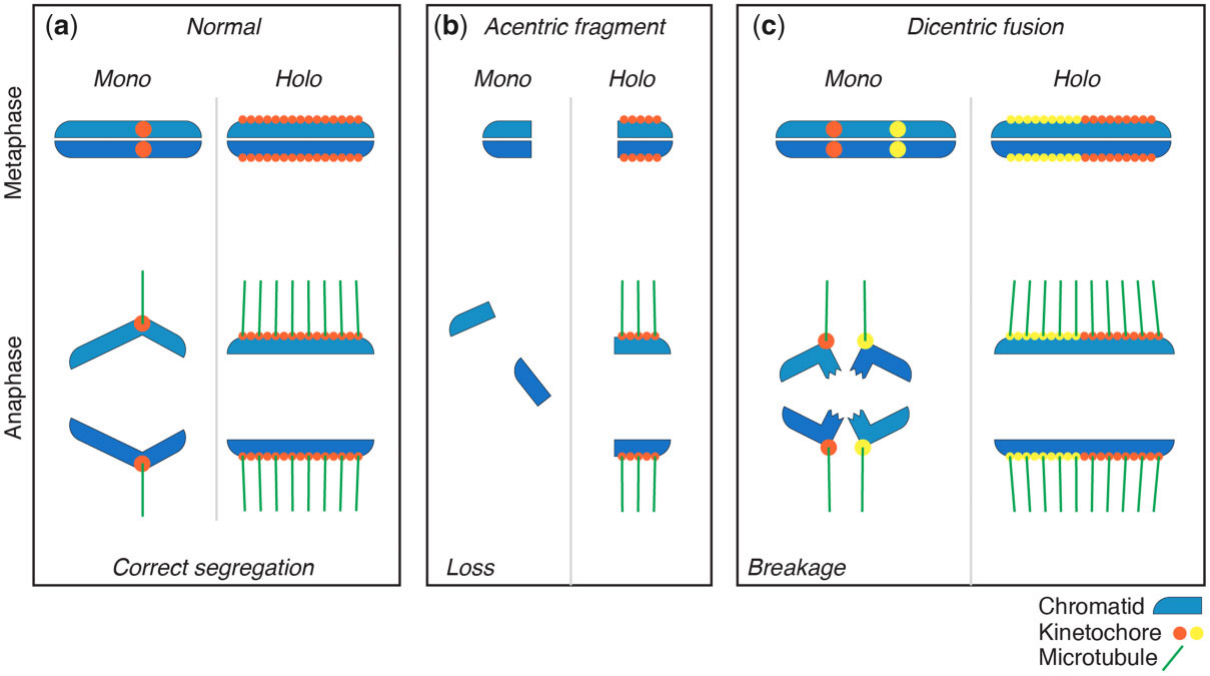
Comparisons of monocentromeres vs holocentromeres in mitotic disjunction. a) Chromosomes with monocentromeres (left) are pulled poleward by the centromere region only, while the rest of the chromosome lags behind; chromosomes with holocentromeres (right) are pulled along their entire lengths and move lengthwise toward the poles. b) Double-strand break damage is especially dangerous to organisms with monocentromeres, since it can result in loss of the entire acentric chromosome fragment distal to the break (left), whereas chromosome fragments with holocentromeres segregate as whole chromosomes (right). c) Fusion of 2 chromosomes with monocentromeres results in dicentrics (left) that can be pulled to opposite poles of the mitotic spindle and break in anaphase, whereas holocentric chromosome fusions segregate as usual (right).

Centromeres play an essential role in chromosome segregation during cell division. Most eukaryotes that have been cytologically examined have monocentric chromosomes, with a single centromere that appears as a primary constriction at metaphase. Early studies of parasitic nematodes found that their chromosomes lack constrictions and orient with their long axes perpendicular to the spindle during mitotic segregation, suggesting spindle attachments along the entire length of the chromosome (Fig. 4). Such chromosomes were described as having holokinetic activity (being pulled everywhere along their length) and were subsequently called holocentric chromosomes. Holocentric chromosomes are found in several plant and animal taxa; among animals, they have been found only in 2 invertebrate phyla: arthropods and nematodes. Electron micrographic studies of a plant-parasitic nematode were the first to show diffuse kinetochores along the length of spermatogonial chromosomes (Goldstein and Triantaphyllou 1980). Detailed EM studies were subsequently carried out on *C. elegans* (Albertson and Thomson 1982, 1993) and then on *Parascaris* (Goday *et al.* 1985, 1992; Goday and Pimpinelli 1986, 1993) characterizing the ultrastructural properties of holocentric chromosomes. Initial studies on mitotic chromosomes demonstrated a trilaminar kinetochore structure consisting of electron-dense inner and outer layers and an electron-lucent middle layer that extended along the length of the entire poleward face of each chromatid (Albertson and Thomson 1982). In spite of their diffuse appearance, more recent EM studies using high-pressure freezing and freeze substitution indicate that the kinetochores of *C. elegans* resemble those of monocentric chromosomes, with a clear zone that excludes ribosomes and other cytoplasmic components along each poleward face of mitotic chromatids and a line of lightly stained material between the clear zone and the chromatin (Howe *et al.* 2001; McIntosh *et al.* 2013). Thus, the overall structural organization of holocentric kinetochores may not be drastically different from monocentric kinetochores.

Holocentric chromosomes are characterized by highly dispersed centromeres and associated kinetochores that assemble on many spatially distinct DNA sequences distributed along the length of each chromosome. The kinetochores interact with proteins and microtubules that drive chromosome segregation during mitosis. Therefore, radiation-induced chromosome breaks do not necessarily lead to chromosome loss in mitosis, since there is no such thing as an acentric fragment (Fig. 4). Centromeres in most organisms are characterized by nucleosomes that incorporate a specialized histone H3 variant (CENP-A or CenH3). CENP-A propagates centromeres epigenetically and facilitates the assembly of the associated kinetochore, the protein complex that connects chromosomes to spindle microtubules (Carroll and Straight 2006).

The *C. elegans* kinetochore contains conserved components observed in other model systems, but the complexity of the kinetochore may be reduced compared to some other organisms. Studies of the mitotic kinetochore in *C. elegans* have recently been reviewed (Pintard and Bowerman 2019) and will not be discussed here. Immunohistochemistry of *C. elegans* mitotic chromosomes indicates that CENP-A is distributed along the length of condensed chromosomes during mitosis and throughout the cell cycle (Buchwitz *et al.* 1999; Oegema *et al.* 2001). Repetitive sequences are typically associated with monocentric centromeres of CENP-A (Janssen *et al.* 2018). However, there does not appear to be an association of repetitive sequences with CENP-A and holocentromeres in *C. elegans*, which is both fascinating and useful as it allows propagation of almost any injected DNA sequence. ChIP-seq analysis of CENP-A along *C. elegans* chromosomes indicated that CENP-A diffusely occupies ∼2,900 broad, low-density domains of ∼10–12 kb that cover about half of the genome (Gassmann *et al.* 2012). CENP-A is nearly completely removed and re-established in chromatin during *C. elegans* embryonic cell divisions (Gassmann *et al.* 2012). Thus, pre-existing CENP-A nucleosomes appear not to be directly required for ongoing deposition of CENP-A. In addition, no specific sequences appear associated with regions of CENP-A incorporation, and neocentromeres can readily be formed on plasmids composed solely of exogenous DNA, suggesting that centromere activity has little or no dependence on underlying sequence (Yuen *et al.* 2011).

Interestingly, *C. elegans* CENP-A incorporation is inversely correlated with genes transcribed in the maternal germline and in early embryos (Gassmann *et al.* 2012). The epigenetic mechanism that regulates changes in kinetochores and CENP-A localization is currently unknown but could involve *C. elegans* proteins that promote CENP-A deposition, including the Myb-domain protein KNL-2 and the histone chaperone LIN-53 (Maddox *et al.* 2007; Lee *et al.* 2016). A historical and broader perspective on holocentric chromosomes can be found below in the second part of this review that concerns other nematodes.

### Telomeres

**Fig. 5.**
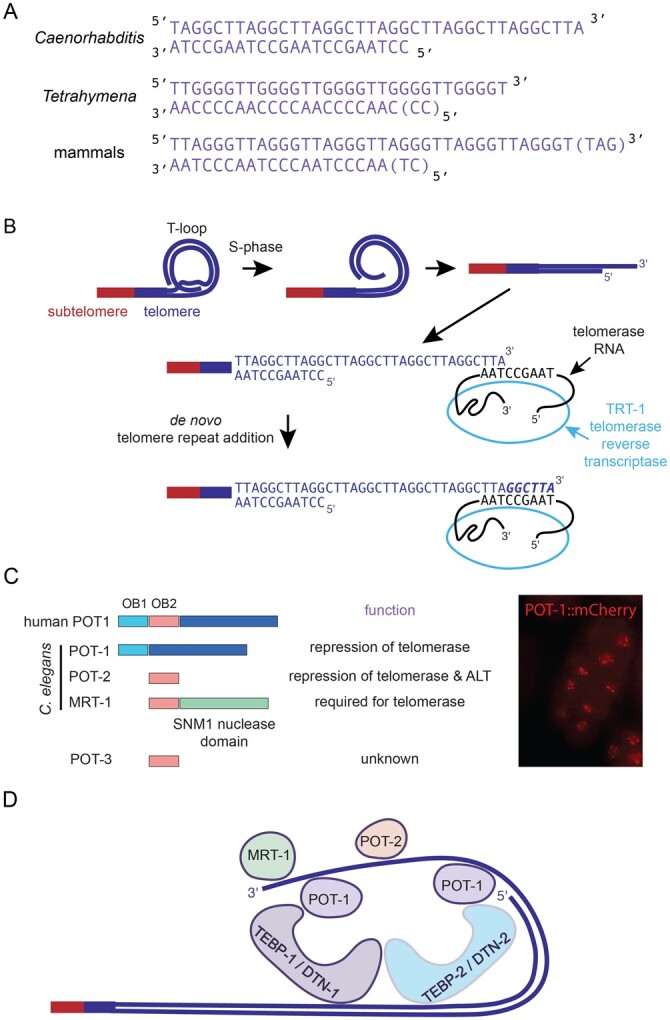
Nematode telomeres. a) Predicted telomere structures from different systems. b) 3′ overhangs of *C. elegans* telomeres may be protected as T-loops that unfold during S-phase, when the 3′ overhang can be extended by telomerase reverse transcriptase. Telomerase RNA template is predicted. c) *Caenorhabditis elegans* Pot1 homologs either limit or are required for telomerase activity (left) and can form foci at telomeres (right). d) Double-stranded DNA telomere-binding proteins TEBP-1/DTN-1 and TEBP-2/DTN-2 interact with one another and with the POT-1 single-stranded telomere-binding protein. POT-2 may interact with POT-1, and MRT-1 may interact with the chromosome terminus in the context of telomere repeat addition.

Eukaryotic chromosome termini are generally composed of tens to thousands of base pairs of repetitive double-stranded telomeric DNA that terminate with a short single-stranded 3′ overhang that is less than 50 nucleotides in length (de Lange 2004). Telomeres consist of a guanine-rich strand that runs 5′ to 3′ toward the chromosome terminus and are typically composed of simple repeat units like (TTGGGG)_n_ in *Tetrahymena* and (TTAGGG)_n_ in mammals (Fig. 5a). Isolation of a somatic telomere created by programmed DNA elimination in the parasitic nematode *Ascaris suum* revealed the telomere repeat telomere repeat sequence (TTAGGC)_n_ (Muller *et al.* 1991; see “*Programmed DNA Elimination*” section below). *Caenorhabditis* *elegans* telomeres were identified by selective cloning of telomeric restriction fragments and associated subtelomeric DNA that is generally not repetitive(Wicky *et al.* 1996) with the exception of tandem repeats at the right subtelomere of Chromosome I and both subtelomeres of Chromosome IV (Wicky *et al.* 1996). Most telomeric clones terminated with the nucleotides 5′-CTTAGG-3′ (Wicky *et al.* 1996), which likely represents the terminal nucleotides of double-stranded telomeric DNA (Fig. 5a). *C. elegans* genomic DNA possesses canonical 3′ and unusual 5′ telomeric overhangs (Cheung *et al.* 2004; Raices *et al.* 2008). Below, we summarize what has been broadly learned about telomere maintenance and dynamics in nematodes.

Most organisms maintain telomere length via telomerase, a ribonucleoprotein that reverse transcribes de novo telomere repeats on to chromosome termini using a template sequence in the noncoding RNA subunit of telomerase (Collins 2006; Fig. 5b). The *C. elegans* telomerase reverse transcriptase TRT-1 was identified in genetic screens for mutations that induce progressive sterility over generations (Meier *et al.* 2006), but the telomerase RNA has yet to be identified in nematodes (Stricklin *et al.* 2005).

Mammalian telomeres are coated with a complex of 6 proteins, termed Shelterin (de Lange 2018). The shelterin subunit POT1, which interacts with single-stranded telomeric DNA via 2 OB-folds, has 4 distinct homologs in *C. elegans* (Fig. 5c). Both POT-1 and POT-2 can promote T-loop formation *in vitro* (Raices *et al.* 2008) and repress telomerase activity in vivo in *C. elegans* (Shtessel *et al.* 2013), whereas the MRT-1 nuclease is required for telomerase activity (Meier *et al.* 2009). A fourth POT1 homolog, POT-3, is encoded by a gene that is attractively adjacent to the right telomere of chromosome III but has no known function (Lowden *et al.* 2008). These distinct *C. elegans* POT1 homologs may reflect the multifunctional nature of POT1 proteins in species like humans and ciliates that possess a single POT1 protein (Baumann and Price 2010).

Proteins that interact with double-stranded telomeric DNA in yeast (Rap1) or in mammals (TRF1 or TRF2) are not apparent in nematode genomes. However, 2 *C. elegans* double-stranded telomere-binding proteins TEBP-1 and TEBP-2 were recently identified based on their affinity for oligonucleotides whose sequence and 3′ overhang structure mirror *C. elegans* telomeres (Fig. 5d; Dietz *et al.* 2021). Reassuringly, all 3 previously defined single-stranded telomere-binding proteins, POT-1, POT-2, and MRT-1, were identified using this approach. TEBP-1 and TEBP-2 proteins were independently identified as DTN-1 and DTN-2, respectively, based on physical interactions with POT-1 (Yamamoto *et al.* 2021). Dysfunction of either TEBP-1 or TEBP-2 results in viable *C. elegans* strains with long or short telomeres, respectively. In contrast, *tebp-1; tebp-2* double mutants become sterile within one or several generations (Dietz *et al.* 2021; Yamamoto *et al.* 2021), suggesting that these paralogous double-stranded telomere-binding proteins redundantly promote an essential cellular function, potentially telomere capping. Many nematodes closely related to *C. elegans* possess a single TEBP homolog, an observation that was elegantly confirmed with protein extracts from *Caenorhabditis* *briggsae* (Dietz *et al.* 2021). TEBP-1/DTN-1 and TEBP-2/DTN-2 possess little or no homology to double-stranded telomere-binding proteins from fungi and mammals (Dietz *et al.* 2021; Yamamoto *et al.* 2021). Consequently, marked changes may have occurred to an ancestral telomere-binding protein during evolution of nematodes or a distinct DNA-binding protein could have been recruited to function at nematode telomeres. Future studies of nematode telomere-binding proteins may provide insight into telomeric and less well-understood nontelomeric roles of telomeres in cell and developmental biology.

Telomere length in the Bristol N2 laboratory strain of *C. elegans* is ∼1–4 kb (Wicky *et al.* 1996; Raices *et al.* 2008). The genomes of 108 wild *C. elegans* strains possess a mean telomere length of 12.5 kb, which results from a subset of strains with very long telomeres (Raices *et al.* 2005; Cook *et al.* 2016). Several wild *C. elegans* isolates with long telomeres possess an F68I *pot-2* mutation (Cook *et al.* 2016). Although this could suggest that POT-2 could be under natural selection, no effect of the F68I isoform of POT-2 or of long or short telomeres has been observed on *C. elegans* fitness in the laboratory (Raices *et al.* 2005; Meier *et al.* 2006; Cook *et al.* 2016).

Although telomerase is expressed in somatic cells of small vertebrates like the mouse, telomerase is silenced in somatic cells of many large vertebrates, including humans, which creates a biological clock that results in gradual telomere shortening. This powerful tumor suppressor mechanism will induce senescence (cell cycle arrest) of cells that proliferate inappropriately. Rare cells that escape cell cycle arrest experience further telomere erosion, resulting in critically shortened telomeres that can become fused with uncapped ends of a sister chromatid or a distinct chromosome. The resulting dicentric end-to-end chromosome fusions often break during cell division to create genome rearrangements that commonly occur during human tumor development. Because nematodes have holocentric centromeres, end-to-end chromosome fusions that arise in *C. elegans* telomerase mutants are stable and can be isolated genetically and studied (Ahmed and Hodgkin 2000; Lowden *et al.* 2008). Chromosome fusions from *C. elegans* telomerase mutants revealed that segments of subtelomeric DNA are commonly copied onto uncapped telomeres by a promiscuous DNA synthesis process (Lowden *et al.* 2011), which contrasts with a long-standing hypothesis that uncapped telomeres might be fused by simple end-joining (McClintock 1941).

**Fig. 6.**
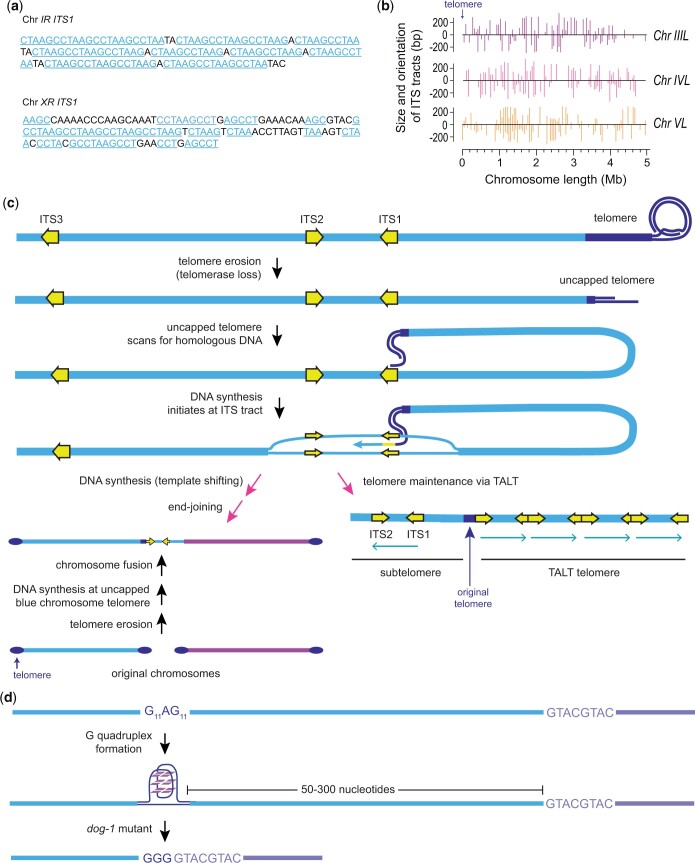
Chromosomal dynamics mediated by telomeric and guanine-rich repetitive DNA. a) ITS tracts at the right ends of *C. elegans* chromosomes I and X. Light blue text highlights 3 or more nucleotides of perfect telomere sequence. b) ITS tracts on the left arms of *C. elegans* chromosomes III, IV, and V. Length of tracts is depicted. ITS tracts that are oriented toward the chromosome terminus are positive lines above the chromosome axis, whereas those that face away from the chromosome terminus are negative lines. c) Functions of ITS tracts. In the absence of telomerase, a telomere (dark blue) becomes critically short and initiates DNA synthesis at an ITS tract (yellow). Right pink arrow: induction of the TALT telomere maintenance pathway if 2 ITS tracts as well as subtelomeric DNA between these tracts (turquoise arrows) are amplified and added to all telomeres. Left pink arrows: blue and purple chromosomes fuse in response to telomere erosion. DNA synthesis initiates at an ITS tract to create a subtelomeric duplication that bridges the fused chromosomes. d) A homopolymeric guanine tract may create a G quadruplex structure that induces deletions of (most of) the guanine tract as well as 50–300 bp of adjacent DNA.

About 15% of *trt-1* telomerase mutants can survive via the telomerase-independent telomere maintenance pathway termed alternative lengthening of telomeres (ALT), which is active in ∼10% of human tumors (Cheng *et al.* 2012; Pickett and Reddel 2015). Most *C. elegans* telomerase mutant strains that survive via ALT display only 3 or 4 chromosomes (Cheng *et al.* 2012), suggesting that a trigger or byproduct of chromosome fusion induces ALT. The POT-2 single-stranded telomere-binding protein represses addition of canonical (TTAGGC)_n_ telomere repeats to chromosome ends via ALT (Cheng *et al.* 2012). A distinct form of ALT, termed Template for ALT (TALT) occurs when interstitial telomere sequence (ITS) tracts that are adjacent to one another and intervening unique sequence DNA are copied to all telomeres of *C. elegans* telomerase mutants (Fig. 6c, lower right; Seo *et al.* 2015). The *C. elegans* genome has 1,229 ITS tracts (Frenk *et al.* 2019), which are degenerate telomere tracts that are plentiful on metazoan chromosome arms (Fig. 6, a and b; Meyne *et al.* 1990; Lin and Yan 2008). About 5% of uncapped telomeres of *C. elegans* telomerase mutants initiate DNA synthesis at ITS tracts, which creates simple or complex subtelomeric duplications that are present at end-to-end chromosome fusion breakpoints (Fig. 6c, lower left; Lowden *et al.* 2011). Together, these studies suggest that the class of repetitive DNA termed ITS tracts has at least 2 distinct biological functions that become apparent in response to telomere dysfunction. Analysis of *Caenorhabditis* nematode genomes suggests that ITS tracts are unlikely to arise in response to stochastic head-to-head telomere fusion events (Frenk *et al.* 2019), so the abundance of ITS tracts on metazoan chromosome arms remains a mystery.

### Repetitive DNA

Nonunique sequences like ITS tracts are abundant in metazoan genomes. Although their repetitive nature can make them challenging to study, unique single nucleotide polymorphisms often allow for analysis of repeat-associated RNA or DNA. For example, extrachromosomal circular DNA is an abundant component of the nuclear DNA in *C. elegans*, *Drosophila* and mammals (Gaubatz 1990; Cohen *et al.* 2003) and is enriched for repetitive sequences that include telomere repeats and repetitive genes like rDNA (Shoura *et al.* 2017). Extrachromosomal circular DNA could reflect a DNA intermediate that might be relevant to the plasticity of repetitive genome sequences.

Telomeres in many organisms display a bias such that the strand running 5′ to 3′ toward the end of a chromosome is G-rich (Fig. 5a). Oligonucleotides containing 4 repeats of the G-rich telomere sequences from *Tetrahymena* (TTGGGG)_4_, *Oxytricha* (TTTTGGGG)_4_, and mammals (TTAGGG)_4_ have been previously shown to fold back on themselves to form 4-stranded DNA structures termed G quadruplexes that are highly stable (Fig. 6d), with in vitro melting temperatures of greater than 80°C (Bochman *et al.* 2012). G quadruplexes are likely to form at telomeres when single-stranded telomeric DNA occurs during DNA replication, but the functional significance of G quadruplexes anywhere in the genome has been a long-standing enigmatic and controversial topic. One clue that G quadruplexes might create toxic structures during DNA replication was discovered for tracts of guanines that experience deletions when the *C. elegans* helicase DOG-1 is deficient (Cheung *et al.* 2002). Although DOG-1 functions in DNA crosslink repair (Youds *et al.* 2008), it appears to be the sole protein in the *C. elegans* genome that promotes guanine tract stability (Kruisselbrink *et al.* 2008). Therefore, homopolymeric tracts of guanines like G_23_ or G_11_AG_11_, which are scattered throughout *C. elegans* chromosomes, form structures that can be impassable to replisomes when DOG-1 is absent, thereby triggering deletion of (most of) the guanine tract as well as 50–300 bp of adjacent DNA (Fig. 6d;Koole *et al.* 2014). These data provide some of the finest evidence for the existence and biological relevance of G quadruplex structures in vivo.

As telomere sequences from various organisms can form highly stable G quadruplexes in vitro, some deletions that occur in *C. elegans dog-1* mutants might be bordered by telomere repeats from *C. elegans* present at one end of the deletion or, rarely, from a short telomere repeat sequence of another organism that stochastically occurs in the *C. elegans* genome that has been shown to form G quadruplexes in vitro. Instead, deletions identified in *dog-1* mutant genomic DNA are almost all flanked by homopolymeric tracts of guanine (Fig. 6d) rather than by 4 repeats of any telomere sequence (Kruisselbrink *et al.* 2008; Koole *et al.* 2014). This suggests that homopolymeric tracts of guanine are able to adopt a highly stable toxic DNA structure that may be distinct from G quadruplex structures formed by telomeric sequences.

Of about 1,700 guanine tracts with the potential to form quadruplex structures in the *C. elegans* genome, 8 were associated with deletions present in the genome of the Hawaiian *C. elegans* strain CB4856 (Koole *et al.* 2014). Three more closely related *C. elegans* genomes CB4857, RC301, and AB2, contained a total of 12 guanine tract-associated deletions. These unexpected results indicate that tracts of guanine-rich DNA guide changes to wild nematode genomes, in the presence of wild-type DOG-1.

Local expansions of paralogous genes can result in tens or hundreds of copies of a gene family whose coding sequences may evolve to promote speciation (International Helminth Genomes Consortium 2019). However, most types of repetitive DNA are composed of “non-coding” segments of the genome that include simple dispersed repeats with 1–20 nucleotide repeat units, tandem satellite repeats composed of 20–180 bp repeat units, and transposons or transposon-derived sequences. Nematode transposons include DNA transposons with terminal inverted repeats that replicate via a cut-and-paste mechanism, helitron transposons that contain hairpins at their 3′ ends and replicate via a rolling circle replication mechanism, and non-LTR retrotransposons that contain poly(A) tails and are reverse transcribed directly onto single-stranded TTTT segments of the genome that are created by a transposon-encoded nuclease. In contrast, LTR retrotransposons have direct long terminal repeats at both ends and are reverse transcribed in the cytoplasm before integration into genomic DNA. LTR retrotransposons are flanked by identical LTR sequences at their termini, although genomes are littered with “solo LTRs” that arise when double-strand breaks within an LTR retrotransposon are repaired by single-strand annealing of flanking LTRs. Additional transposon byproducts found in nematode genomes include long tandem repeat tracts that can be created by the rolling circle replication mechanism of helitron transposons (Feschotte and Pritham 2007; Garrigues *et al.* 2019).

Transposons are normally silenced in germ cells in order to limit the damage that transposition can wreak on genomes (Castel and Martienssen 2013; Ozata *et al.* 2019). Although transposon movement does not occur in Bristol N2 *C. elegans* germ cells under standard laboratory conditions (Eide and Anderson 1985), high levels of transposon movement in germ cells were initially observed in lab experiments with the wild *C. elegans* strain Bergerac (Emmons *et al.* 1983; Liao *et al.* 1983). These surprising results suggest that persistent defects in transposon silencing may not compromise nematode survival in the wild. Transposon tagging was an attractive method of gene identification during the pregenomic era, and a genetic screen defined *mutator* mutants that display transposon movement in germ cells, typically of Tc1 and Tc3 DNA transposons (Ketting *et al.* 1999; Bessereau 2006). Some *mutator* mutants are deficient for both transposon silencing and for gene silencing in response to exogenous dsRNAs, whereas others are deficient for an as yet undescribed process that recognizes transposon transcripts as foreign or aberrant RNA and targets them for small RNA biogenesis (Sijen and Plasterk 2003).

**Fig. 7.**
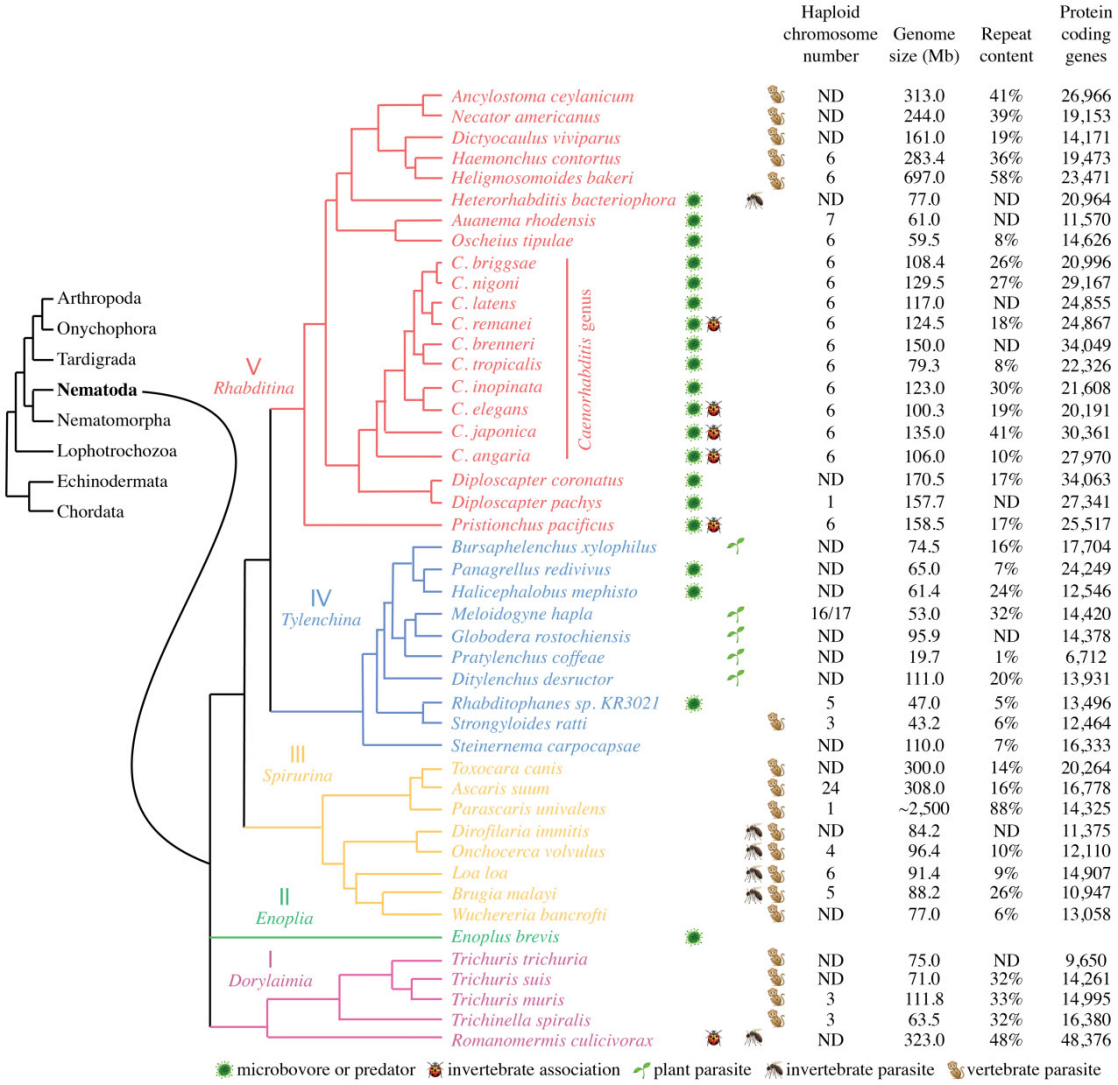
A composite cladogram with local details taken from different studies depicts the phylogenetic structure of some nematode species with sequenced genomes. The overall structure of the clades is based on nuclear small subunit ribosomal RNA analyses and interpretation of taxon relationships derived from morphology. Taxon systematic names are given for the major nodes in the phylogeny. Clades I, II, III, IV, and V were first defined in Blaxter *et al.* (1998). This cladogram shows predicted relationships between species based on comparisons of several contemporary phylogenies (Blaxter and Koutsovoulos 2015; Tian *et al.* 2015; Haag *et al.* 2018; McLean *et al.* 2018; International Helminth Genomes Consortium 2019). The ecosystem and trophic habits are indicated by small icons, as defined at the bottom of the diagram. Some parasites that reproduce in vertebrates spend stages of their lifecycle in invertebrate hosts, as indicated by invertebrate parasite icons [from Blaxter and Koutsovoulos (2015)]. For *Ascaris* and *Parascaris*, germline chromosome number and DNA content are shown (see Table 1). ND: not determined or conflicting data.

Analysis of a wild *C. elegans* strain has revealed that the Cer1 LTR retrotransposon can move to inactivate a gene that represses copulatory plug formation (Palopoli *et al.* 2008). To comprehensively assess how commonly transposons move in wild nematodes, the genomes of 152 wild *C. elegans* strains with distinct genome sequences (haplotypes) were studied and found to possess 385 transposon insertions that were present in a recent common ancestor of extant *C. elegans* strains (Cook *et al.* 2016; Laricchia *et al.* 2017). The 152 genomes contained 2,771 unique transposon insertion sites, with an average number of 72 novel insertions per strain, including DNA transposons Tc1, Tc3, Tc5, MARINER2, and MIRAGE1 and retrotransposons CELE45, LTRCER1, and CER2-1 (Laricchia *et al.* 2017). The abundant evidence for transposition events in wild *C. elegans* strains implies that endogenous transposon silencing pathways are occasionally repressed, either by mutation of transposon silencing genes as seen for Bergerac or by a form of environmental stress that remains to be understood. Alternatively, discrete bursts of transposition could occur when a fragment of foreign genomic DNA that contains an exogenous transposon is introduced into a naive germ cell nucleus via horizontal gene transfer (see Extrachromosomal Array section below; Shen *et al.* 2020). Novel transposon insertions may cause changes to gene expression or structure that contribute to fitness in the context of evolutionary change (Garrigues *et al.* 2019; Webster *et al.* 2019). Consistent with a role for transposons in speciation, the larger genome of the closest known relative of *C. elegans*, *Caenorhabditis* *inopinata* (Fig. 7), partially results from dramatic expansion of endogenous LTR retrotransposons and the Tc1 class of DNA transposon (Kanzaki *et al.* 2018; Woodruff and Teterina 2020).

Similar to guanine tracts that are associated with spontaneous deletions that occur regularly in *dog-1* mutants (Koole *et al.* 2014), transposons represent another form of repetitive DNA that commonly alter genomes of wild *C. elegans* strains (Laricchia *et al.* 2017). In addition, analysis of genomes of wild *C. elegans* strains suggests that chromosome evolution may be shaped by TALT events at ITS tracts that occur in the presence of telomerase (Kim *et al.* 2019). These independent examples indicate that changes to repetitive DNA that occur in *C. elegans* genome stability mutants grown in laboratories can be generally relevant to understanding of how chromosomes of wild nematodes and other organisms evolve. Many open questions remain about how the complex and rapidly changing repertoire of repetitive DNA in nematode genomes shapes gene expression and evolutionary plasticity (Garrigues *et al.* 2019).

### Extrachromosomal arrays

Microinjection of linear or supercoiled double-stranded DNA into the syncytial cytoplasm of *C. elegans* meiotic germ cells can lead to creation of structures termed extrachromosomal arrays whose electrophoretic mobility resembles that of uncut *C. elegans* genomic DNA (Stinchcomb *et al.* 1985; Mello *et al.* 1991). Microinjection of a low-abundance plasmid with an 100-fold molar excess of carrier DNA (either another plasmid or fragmented N2 wild-type genomic DNA) typically results in extrachromosomal arrays that contain several copies of the low-abundance plasmid (Mello *et al.* 1991). Consistently, extrachromosomal arrays are detected as small DAPI-positive spots in mature oocyte nuclei (Stinchcomb *et al.* 1985), with estimated sizes of 1–13 Mb (Stinchcomb *et al.* 1985; Yuen *et al.* 2011; Woglar *et al.* 2020; Lin *et al.* 2021). Together, these data suggest that extrachromosomal arrays are composites of hundreds of plasmid molecules.

After microinjection of plasmids into adult hermaphrodite germ cells, extrachromosomal arrays form in the newly fertilized zygotes produced by the injected mothers (Yuen *et al.* 2011). Therefore, the syncytial cytoplasm of adult germ cells may lack one or more nuclear factors needed to promote extrachromosomal array formation. Centromere formation lags behind extrachromosomal array assembly, as extrachromosomal arrays fail to segregate accurately during mitosis until several embryonic cell divisions have been completed (Yuen *et al.* 2011). One consequence of this segregation defect is that F1 animals that express array marker genes in their somatic cells are frequently mosaic and fail to transmit extrachromosomal arrays to F2 progeny. However, arrays that are transmitted to the F2 generation can often be propagated indefinitely, consistent with acquisition of a stable centromeric state.

Extrachromosomal arrays created from identical solutions of injected DNA are transmitted between generations at highly variable rates from one generation to the next, ranging in frequency from less than 5% to greater than 90%. However, individual extrachromosomal arrays display relatively stable levels of transmission. The remarkable variability in transmission of distinct extrachromosomal arrays suggests substantial heterogeneity in their genetic or epigenetic structures. Although telomeres can be required for the stability of artificial chromosomes (Allshire 1995; Cooke 2001), stable *C. elegans* extrachromosomal arrays have circular structures and can be established in the absence of telomerase, implying that they lack telomeres (Meier *et al.* 2006; Woglar *et al.* 2020). Given the importance of telomere addition to artificial chromosomes in other experimental systems, it is surprising that plasmids containing natural *C. elegans* telomeres have not yet been tested for the ability to stimulate creation of linear extrachromosomal arrays in nematodes (Wicky *et al.* 1996).

A role for homologous recombination in extrachromosomal array formation was elegantly established by injection of reporter gene fragments that possessed 300 or 600 base pairs of identity (Mello *et al.* 1991). Plasmid DNA prepared for microinjection is often supercoiled or nicked, which can expose single-stranded plasmid segments that could facilitate homologous recombination. Moreover, *E. coli* plasmids can contain significant segments of identity, such as antibiotic resistance genes, which could facilitate inter plasmid recombination. However, homologous recombination does not occur for DNA fragments that are microinjected with a substantial excess of carrier DNA (Lin *et al.* 2021).

Microinjection of linear plasmid DNA results in extrachromosomal arrays with plasmids in head-to-tail, head-to-head, and tail-to-tail orientations (Stinchcomb *et al.* 1985), and array biogenesis depends on homologous recombination (RAD-51) and nonhomologous end-joining (LIG-4; Kemp *et al.* 2007; Yuen *et al.* 2011). A third DNA repair pathway, microhomology-mediated end-joining, promotes error-free recombination for linear DNA fragments with only 10 base pairs of terminal identity (Kemp *et al.* 2007). Insight into biogenesis of extrachromosomal arrays has been gleaned from high-throughput sequence analysis of an extrachromosomal array created using fragments of *Saccharomyces* *cerevisiae* DNA, which revealed that DNA fragments that are greater than 1 Kb in length were preferentially targeted for assembly into extrachromosomal arrays (Lin *et al.* 2021).

Although many extrachromosomal arrays can stably express transgenes in somatic cells, silencing of transgenes in germ cells often occurs within several generations (Kelly *et al.* 1997). Array silencing may be triggered by the variable orientations of plasmids and promoters in extrachromosomal arrays, which may occasionally produce hairpin RNAs that trigger small RNA biogenesis and gene silencing in germ cells (Hedgecock and Herman 1995; Tavernarakis *et al.* 2000). Transgene silencing or cosuppression occurs in germ cells of many metazoans, including *C. elegans*, reflecting a small RNA silencing response likely triggered by hairpin or sense or antisense transcripts and antisense transcripts that emanate from complicated gene structures within extrachromosomal arrays (Dernburg *et al.* 2000; Ketting and Plasterk 2000). Germline silencing of extrachromosomal arrays can be suppressed by diluting injected plasmid DNA 1:100 with restriction fragments of *C. elegans* wild-type genomic DNA, which may limit palindrome assembly during array creation (Kelly *et al.* 1997). Moreover, about one-tenth of the *C. elegans* genome is composed of a repetitive DNA element termed Periodic A_n_/T_n_ Clusters (Fire *et al.* 2006). Remarkably, addition of Periodic A_n_/T_n_ Clusters to introns of fluorescent epitope-tags can promote germline gene expression, even for transgenes present in repetitive extrachromosomal arrays that normally evoke a potent germline silencing response (Aljohani *et al.* 2020). Therefore, Periodic A_n_/T_n_ Clusters likely help to mark nematode chromosome segments as self DNA. The CSR-1 Argonaute protein promotes a small RNA pathway that promotes expression of endogenous *C. elegans* genes in germ cells and embryos (Seth *et al.* 2013; Wedeles *et al.* 2013, 2014), and it is plausible that CSR-1 could interact directly with or act in parallel to Periodic A_n_/T_n_ Cluster repeats to promote gene expression.

If *C. elegans* strains that possess repetitive extrachromosomal arrays are thawed and re-tested, published results can be challenging to reproduce. It is possible that changes to the genetic or epigenetic structures of extrachromosomal arrays could contribute to the variable nature of some published results (Klosin *et al.* 2017). In response to such concerns, transposons or nucleases that induce site-specific DNA double-strand breaks have been developed to create single-copy transgene insertions or to modify endogenous genes. Given the ease with which extrachromosomal arrays can be generated and advances such as use of Periodic A_n_/T_n_ Clusters to promote expression of genes within repetitive arrays, repetitive extrachromosomal arrays may remain attractive tools for contemporary analysis of nematode biology.

### Chromatin and higher-order organization of chromosomes

DNA segments typically interact with nucleosomes whose histone subunits are modified in a manner that dictates transcriptional regulation and nuclear position. *Caenorhabditis* *elegans* was a pioneering model system in this regard, where modENCODE research groups collaborated to create a detailed map of the 2D landscape of the nucleus (Gerstein *et al.* 2010). Locations of transcription, transcription factors, and histone modifications throughout the genome were mapped at high resolution in an effort to generate an unprecedented high-resolution understanding of genome organization. This collaborative research project validated and utilized antibodies that recognize a number of histone modifications for chromatin immunoprecipitation experiments, which provided detailed insights into global and local chromatin organization in coding and noncoding segments of the genome (Egelhofer *et al.* 2011).

The modENCODE efforts demonstrated that chromosome arms of autosomes are heavily enriched for heterochromatic histone methylation marks that reflect locations of repetitive sequences including tandem repeats and transposons as well as nonfunctional pseudogenes (Gerstein *et al.* 2010). In contrast, central regions of autosomes are repeat-poor and are enriched for constitutively expressed essential genes as well as a distinct class of genes that are silent in germ cells but expressed in the soma. In contrast to autosomes, the X chromosome possesses a more even distribution of genes, repeats, and chromatin marks. Promoters of active genes are associated with histones that are characterized by acetylation of H3K27 and methylation of H3K4 (Brown and Celniker 2015). Genes that are facultatively silenced in germ cells but become expressed during somatic development are decorated in embryos with the H3K27me3 silencing mark created by the MES-2/MES-3/MES-6 PRC2-like silencing complex. Methylation of histone H3K9 and H3K27 creates blocks of constitutive heterochromatin at repeat sequences and pseudogenes scattered across the genome (Ahringer and Gasser 2018). Although methylation of H3K9 and H3K27 is anti-correlated in humans and flies (Ho *et al.* 2014), their patterns overlap extensively in worms, suggesting that facultative and constitutive heterochromatin may not be as distinct as in other experimental systems. The distribution of histone modifications is guided in part by passage of marked histones from parent chromosomes to daughter chromatids and by transcription factors that interact directly with double-stranded DNA to recruit histone-modifying proteins and create a complex local landscape of epigenetic marks (Gaydos *et al.* 2014). Small RNAs can function in the nucleus by interacting with RNA transcripts in a sequence-specific manner that, similar to transcription factors, recruit histone-modifying proteins to promote or repress creation of RNA transcripts (Frolows and Ashe 2021). Once initiated, the process of transcription itself lays down histone modifications that specially mark and identify genes. The inner nuclear lamins interact with ∼⅓ of genomic DNA and help to orchestrate the structure and expression of genomic DNA within the nucleus. Analysis of the nuclear lamina-associated protein LEM-2 revealed interactions of the nuclear envelope with autosome arms and with the left end of the X chromosome (Ikegami *et al.* 2010), which are enriched for regions of the genome that experience transcriptional repression.

The modENCODE consortium determined the binding sites of 57 transcription factors, which revealed low-complexity regions of the genome that interact with few transcription factors via specific sequence motifs to drive gene expression in specific tissues. However, several hundred highly occupied target (HOT) regions that may simultaneously interact with many transcription factors were also identified, even though they lack canonical transcription factor sequence motifs. These HOT regions are close to transcription start sites of housekeeping genes and may promote their transcription in many distinct cell types (Gerstein *et al.* 2010; Van Nostrand and Kim 2013; Kudron *et al.* 2018; Wreczycka *et al.* 2019).

The 3D organization of the genome has been studied using Hi-C technology, where cross-links are created between segments of the genome in close physical proximity, followed by genome fragmentation and high-throughput sequencing, which reveals which fragments are ligated. Hi-C analysis has allowed for models of local and long-range physical interactions within the genome to be created (Evans *et al.* 2016; Brejc *et al.* 2017; Anderson *et al.* 2020). All segments of a single chromosome are readily revealed by Hi-C, due to frequent physical interactions between chromosome sequences that are in close linear proximity. In addition, Hi-C can reveal long-range interactions for more distant segments of a single chromosome or, occasionally, for distinct segments of different chromosomes (Xu and Dixon 2020). Hi-C studies may facilitate an understanding of higher-order chromosome structures within *C. elegans* nuclei that are relevant to genome biology.

## Section 2: Chromosome biology of other nematodes

### Nematode diversity

While the free-living terrestrial microbivore *C. elegans* is the best-studied nematode, nematodes are a diverse phylum adapted to a wide variety of lifestyles (De Ley 2006). They range in size from ∼1 mm (most free-living nematodes) to as large as 8 m (*Placentonema* *gigantissima* in sperm whales) and are found in almost all ecosystems. While most are free-living, many are parasites of plants or animals. Estimates suggest that nematodes represent ∼80% of all metazoans and thus are the most abundant animals on Earth (Eisenhauer and Guerra 2019; van den Hoogen *et al.* 2019). As nematodes are highly diverged from each other and have substantial variation in their genes, pathways, and molecular processes, studies that have focused on *C. elegans* chromosomes may not be representative of the nematode phylum as a whole.

Phylogenetic analyses have led to the division of nematodes into 3 major classes and 5 clades of nematodes: Dorylaimia (Clade I), Enoplia (Clade II), and the 3 clades of Chromadoria (Spirurina—Clade III, Tylenchina- Clade IV, and Rhabditina—Clade V) (see Fig. 7) (Blaxter *et al.* 1998; van Megen *et al.* 2009; Blaxter and Koutsovoulos 2015; Smythe *et al.* 2019). *Caenorhabditis* *elegans* and its relatives are members of Clade V, which also includes the model system *Pristionchus pacificus* (Sommer and McGaughran 2013). Genomic and other information (see Table 1) is available for Clade IV largely from animal and plant parasites (e.g. *Strongyloides*, *Heterodera*, *Meloidogyne*, and *Pratylenchus*), for Clade III from human and other animal parasites (e.g. *Ascaris* spp., *Brugia* spp., and *Oncocherca* spp.), and for Clade I from animal and plant parasites (e.g. *Trichinella*, *Trichuris*, *Romanomermis*, and *Xiphinema*). Very little information is currently available for Clade II, which like Clade I is considered a more basal nematode clade.

**Table 1.** Selected genomes and information.

Nematode	Lifestyle	Clade	Genome Browser	N50	Genome Assembly or Size in Mb	Coding Gene Number	Repeat Content	Germline Haploid Chromosome Number	Sex Determination System	Reproductive mode
*Acrobeloides nanus*	Bacteriovore	Clade IV	JBrowse | Ensembl	19,572	248.0	32,984	53%	ND	ND	
*Ancylostoma ceylanicum*	Animal parasite (mammalian hookworm)	Clade V	JBrowse | Ensembl	668,412	313.0	26,966	41%	ND	ND	Sexual, gonochoristic/dioecious
*Auanema rhodensis*	Bacteriovore	Clade V		556,081	61.0	11,570	ND	6A + 1X	XX/XO	Sexual, gonochoristic/hermaphrodite = trioecious
*Ascaris suum*	Animal parasite (pigs and humans)	Clade III	JBrowse | Ensembl	4,646,302	252 (somatic) 308 (germline)	16,778	16%	19A + 5X or 10X (somatic) 1 (germline)	XX/XO	Sexual, gonochoristic/dioecious
*Brugia malayi*	Animal parasite (human filariia)	Clade III	JBrowse | Ensembl	14,214,749	88.2	10,947	26%	4A + X Y	XY	Sexual, gonochoristic/dioecious
*Bursaphelenchus xylophilus*	Plant parasite (pine trees)	Clade IV	JBrowse | Ensembl	949,830	74.5	17,704	16%	5A + 1X	XX/XO	Sexual, gonochoristic/dioecious
*Bursaphelenchus mucronatus*	Plant parasite (pine trees)	Clade IV		1,480,000	73.0	13,696	22%%			
*Caenorhabditis bovis*	Animal associated	Clade V		7,560,000	63.0	13,218	13%			
*Caenorhabditis brenneri*	Bacteriovore	Clade V	JBrowse | Ensembl	377,250	150.0	34,049	n/d	5A + 1X	XX/XO	
*Caenorhabditis briggsae*	Bacteriovore	Clade V	JBrowse | Ensembl	17,485,439	108.4	20,996	26%	5A + 1X	XX/XO	Androdioecious hermaphrodite
*Caenorhabditis elegans*	Bacteriovore	Clade V	JBrowse | Ensembl	17,493,829	100.3	20,191	19%	5A + 1X	XX/XO	Androdioecious hermaphrodite
*Caenorhabditis latens*	Bacteriovore	Clade V	JBrowse | Ensembl	366,678	117.0	24,855	ND	5A + 1X	XX/XO	
*Caenorhabditis nigoni*	Bacteriovore	Clade V	JBrowse | Ensembl	20,390,332	129.5	29,167	27%	5A + 1X	XX/XO	
*Caenorhabditis remanei*	Bacteriovore	Clade V	JBrowse | Ensembl	21,502,000	124.9	26,308	21%%	5A + 1X	XX/XO	
*Caenorhabditis remanei*	Bacteriovore	Clade V	JBrowse | Ensembl	1,522,088	118.5	24,977	18%	5A + 1X	XX/XO	
*Caenorhabditis remanei*	Bacteriovore	Clade V	JBrowse | Ensembl	1,765,890	124.5	24,867	18%	5A + 1X	XX/XO	
*Caenorhabditis sinica*	Bacteriovore	Clade V	JBrowse | Ensembl	25,151	132.0	34,696	19%	5A + 1X	XX/XO	
*Caenorhabditis sp34 inopinata*	Bacteriovore	Clade V	JBrowse | Ensembl	20,594,552	123.0	21,608	30%	5A + 1X	XX/XO	
*Caenorhabditis tropicalis*	Bacteriovore	Clade V	JBrowse | Ensembl	20,921,866	79.3	22,326	8%	5A + 1X	XX/XO	Androdioecious hermaphrodite
*Dictyocaulus viviparus*	Animal parasite (ruminants)	Clade V	JBrowse | Ensembl	225,234	161.0	14,171	19%	5A + 1X	XX/XO	
*Diploscapter coronatus*	Bacteriovore	Clade V	JBrowse | Ensembl	1,007,652	170.5	34,063	17%	1	ND	Parthenogenetic
*Diploscapter pachys*	Bacteriovore	Clade V	JBrowse | Ensembl	124,169	157.7	27,341	ND	1	ND	Parthenogenetic
*Ditylenchus destructor*	Plant parasite (potato)	Clade IV	JBrowse | Ensembl	555,026	111.0	13 931	20%	ND	ND	
*Enterobius vermicularis*	Animal parasite (human pinworm)	Clade III	JBrowse | Ensembl	20,546	150.0	12,895	ND	ND	ND	Sexual, gonochoristic/dioecious
*Globodera rostochiensis*	Plant parasite (tomato and potato)	Clade IV	JBrowse | Ensembl	88,495	95.9	14,378	ND	ND	ND	
*Haemonchus contortus*	Animal parasite (ruminants)	Clade V	JBrowse | Ensembl	47,382,676	283.4	19,473	36%	5A + X	XX/XO	
*Halicephalobus mephisto*	Bacteriovore (subterranean, elevated Temp.)	Clade IV	JBrowse | Ensembl	313,311	61.4	12,546	24%	ND	ND	Parthenogenetic
*Heligmosomoides bakeri*	Animal parasite (rodents)	Clade V	JBrowse | Ensembl	35,732	697.0	23,471	58%	5A + 1X	ND	
*Heterodera glycines*	Plant parasite (soybeans)	Clade IV	JBrowse | Ensembl	304,127	124.0	29,679	36%	ND	ND	
*Heterorhabditis bacteriophora*	Bacteriovore	Clade V	JBrowse | Ensembl	312,328	77.0	20,964	ND	ND	ND	
*Meloidogyne hapla*	Plant parasite (root-knot)	Clade IV	JBrowse | Ensembl	37,501	53.0	14,420	32%	7	ND	Meiotic parthenogenetic with occasional outcrossing
*Meloidogyne luci*	Plant parasite (root-knot)	Clade IV		1,711,905	209.0					
*Necator americanus*	Animal parasite (human hookworm)	Clade V	JBrowse | Ensembl	211,860	244.0	19,153	39%	ND	ND	
*Oesophagostomum dentatum*	Animal parasite (pigs)	Clade V	JBrowse | Ensembl	19,257	490.0	25,291	31%	ND	ND	
*Onchocerca volvulus*	Animal parasite (human filariia)	Clade III	JBrowse | Ensembl	25,485,961	96.4	12,110	10%	3A + X Y	X/Y	Sexual, gonochoristic/dioecious
*Oscheius tipulae*	Bacteriovore	Clade V	JBrowse | Ensembl	1,203,411	59.5	14,626	8%	5A + X	XX/XO	
*Panagrellus redivivus*	Bacteriovore	Clade IV	JBrowse | Ensembl	262,414	65.0	24,249	7%	ND	ND	
*Parascaris univalens*	Animal parasite (horses)	Clade III	JBrowse | Ensembl	1,825,986	253.4 (somatic) ∼2,500 (germline)	14,325	88%	1	ND	Sexual, gonochoristic/dioecious
*Pratylenchus coffeae*	Plant parasite	Clade IV		10,000	19.7	6,712	1%	ND	ND	
*Pristionchus pacificus*	Omnivore	Clade V	JBrowse | Ensembl	23,915,096	158.5	25,517	17%	5A + 1X	XX/XO	Sexual, gonochoristic/dioecious
*Rhabditophanes sp. KR3021*	Bacteriovore	Clade IV	JBrowse | Ensembl	537,195	47.0	13,496	5%	5A	ND	Meiotic parthenogenesis
*Romanomermis culicivorax*	Entomopathogenic	Clade I	JBrowse | Ensembl	17,583	323.0	48,376	48%	ND	ND	Sexual, gonochoristic/dioecious
*Steinernema carpocapsae*	Entomopathogenic	Clade IV	JBrowse | Ensembl	299,566	84.5	30,957	ND	4A + 1X	ND	
*Steinernema carpocapsae (Breton)*	Entomopathogenic	Clade IV		1,245,171	111.0	16,333	7%%	4A + 1X	ND	
*Strongyloides ratti*	Animal parasite (rats)	Clade IV	JBrowse | Ensembl	11,693,564	43.2	12,464	6%	2A + 1X	XX/XO	
*Toxocara canis*	Animal parasite (dogs)	Clade III	JBrowse | Ensembl	31,084	300.0	20,264	14%	12A + 6X (Germline)	XX/XO	Sexual, gonochoristic/dioecious
*Trichinella spiralis*	Animal parasite (pigs and mammals)	Clade I	JBrowse | Ensembl	6,373,445	63.5	16,380	32%	2A + XX or X0	XX/XO	Sexual, gonochoristic/dioecious
*Trichuris trichiura*	Animal parasite (humans)	Clade I	JBrowse | Ensembl	69,794	75	9,650	ND	ND	ND	
*Trichuris muris*	Animal parasite (mice)	Clade I	JBrowse | Ensembl	28,941,788	111.8	14,995	33%	2A + X Y	X/Y	Sexual, gonochoristic/dioecious
*Trichuris suis*	Animal parasite (pigs)	Clade I	JBrowse | Ensembl	503,034	74.2	14,436	32%	ND	X/Y	Sexual, gonochoristic/dioecious
*Trichuris suis*	Animal parasite (pigs)	Clade I	JBrowse | Ensembl	443,734	71.0	14,261	32%	ND	X/Y	Sexual, gonochoristic/dioecious
*Trichuris suis*	Animal parasite (pigs)	Clade I	JBrowse | Ensembl	1,322,386	63.8	9,831	ND	ND	X/Y	Sexual, gonochoristic/dioecious
*Meloidogyne enterolobii*	Plant parasite (root-knot)	Clade IV	BioRxiv paper	143,000	240.0	59,773	17.5	ND	ND	Mitotic parthenogenetic (triploid)
*Caenorhabditis bovis*	Associated with cattle ears	Clade V	BioRxiv paper	7,600,000	63	13,128	13.7	ND	ND	

N50 = defined as the minimum contig length needed to cover 50% of the genome.

Genome assembly or size in Mb = numbers may be under or over estimates due to the inability to assembly repetitive sequences, heterozygosity, or other aspects of the genome.

Coding Gene Number = estimate of number of coding genes may vary due to overs estimates due to splitting of genes or underestimates from gene characterization software.

Repeat Content = estimate of repeat content in genome typically from read numbers; these estimates are also subject to how repeat sequences are defined.

ND, not available.

### Chromosomes in other nematodes

Nematodes generally have a haploid chromosome number of 4–12 chromosomes, but larger and smaller numbers have been cataloged (Triantaphyllou 1983). All *Caenorhabditis* species investigated so far have a chromosome number of 2*n* = 2x = 12 in the homogametic sex (5 pairs of autosomes and 1 pair of X chromosomes in females or hermaphrodites), while heterogametic males have only a single X chromosome. While genetic sex determination is most common in nematodes, environmental sex determination has been adopted by a number of nematodes including mermithids and *Strongyloides* spp. The most common form of genetic sex determination in nematodes is XX/XO, which may have been ancestral, but Y chromosomes have been found in a small minority of nematodes in Clades II and III (Coghlan 2005).

The holocentric nature of most nematode chromosomes may favor the creation of stable chromosome fusions. In this regard, a few nematode species are known to possess only a single pair of chromosomes: *Diploscapter pachys*, *Diploscapter* *coronatus*, and *P.* *univalens* (Niedermaier and Moritz 2000; Fradin *et al.* 2017; Hiraki *et al.* 2017). The single germline chromosome of *P.* *univalens* breaks during somatic development and telomerase establishes many new (TTAGGC)_n_ telomeres to create an estimated 32 (29 autosomes and 3X in male) or 35 (29 autosomes and 6X in female) haploid chromosomes (Niedermaier and Moritz 2000; see below), so the single germline chromosome of *Parascaris* evolved via a chromosome fusion process that occurred in the presence of telomerase. In contrast, the *D.* *pachys* genome is devoid of long tracts of (TTAGGC)_n_ telomere repeats, suggesting that its single chromosome may have evolved as a consequence of telomerase loss (Fradin *et al.* 2017). 

The single-chromosome nematode species present an interesting special case for chromosomal sex determination. In the soma of *P. univalens*, X chromosomes display sex-specific dosage: females have twice the number of X chromosomes that males have, making the soma a typical XX/XO system. However, since only a single pair of chromosomes exists in the zygote, the possibilities are that either (1) one chromosome of the pair lacks X sequence in males, meaning that the 2 chromosomes are different and male zygotes are effectively XY or (2) both chromosomes are identical, meaning that sex determination is not chromosome-based, and some other factor lead to loss of X sequences and subsequent male development. Combined cytological and genomic approaches should be able to shed light on this question.

### Genomes of other nematodes

Many nematode species have had their entire genomes sequenced due to their importance for health and agriculture (Fig. 7). However, these genome assemblies are typically not as comprehensive as that for *C. elegans*, being primarily constructed based on short-read 50–150 nt next-generation sequencing reads. A few other nematode genomes contain whole-chromosome assemblies (*C. briggsae*, *C. inopinata*, *Caenorhabditis* *nigoni*, *Caenorhabditis* *remanei*, *Pristionchus, Strongyloides, Ascaris*) with gaps (Ross *et al.* 2011; Fierst *et al.* 2015; Hunt *et al.* 2016; Rödelsperger *et al.* 2017; Kanzaki *et al.* 2018; Yin *et al.* 2018; Teterina *et al.* 2020; Wang *et al.* 2020), but most nematode genome assemblies consist of hundreds to thousands of fragments rather than full chromosome assemblies. These genomes nevertheless provide a rich source of information that includes a rich source of predicted genes and proteins (Baptista and Kissinger 2019). Table 1 provides examples of genomes that are well finished (Stevens *et al.* 2020; Susič *et al.* 2020; Teterina *et al.* 2020; Wu *et al.* 2020).

Self-fertile nematodes, including *C. elegans*, have low levels of heterozygosity, which makes them optimal for creation of haploid reference genomes. However, outcrossing nematode species, or those that are parthenogenetic, can possess long genomic intervals that are obligately heterozygous and have highly diverged sequences (Barrière *et al.* 2009; Schwarz 2017). For example, the discovery of a large block of obligate heterozygosity in *C. remanei* that was maintained by balancing selection during inbreeding, caused the initial *C. remanei* reference genome of 150 Mb to be reduced to 124 Mb (Barrière *et al.* 2009; Schwarz 2017). Another concern for chromosomes of outcrossing species is that the genomes of individual animals are very different from one another. These and other difficulties with modern genome sequence analysis can lead to errors in assembly, which likely affect gene and genome size estimates for many of the outcrossing species shown in Fig. 7. Improved genome assemblies will enable analysis of nematode chromosome evolution, genetic diversity, population studies, pathogenicity, and evolution of resistance to anthelminthic compounds (Chin *et al.* 2016; Weisenfeld *et al.* 2017; Garg *et al.* 2018; Dilks *et al.* 2020; Wit *et al.* 2021).

Repetitive DNA is often difficult to incorporate into a genome assembly, leading to gaps and accompanying errors in genome size. Modern long-read sequencing technologies (Baptista and Kissinger 2019) revealed errors in the original Bristol N2 reference *C. elegans* genome, revealing 42 remaining gaps that could not be spanned (Tyson *et al.* 2018). Use of the PacBio long-read sequencing platform (5–200 Kb) that has high error rates in conjunction with short-read and Nanopore sequencing (up to 500 Kb) enabled interrogation of the chromosomes of VC2010, which is a modern-day descendant of the Bristol N2 strain that was used to create the initial *C. elegans* genome assembly. This integrative effort discovered a number of previously undetected segments of the *C. elegans* genome and filled almost all chromosome sequence gaps (Yoshimura *et al.* 2019). Sequencing of 608 wild isolates of *C. elegans* has revealed that about 20% of the genome is highly divergent, which makes these selfing strains as genetically diverse as outcrossing *Caenorhabditis* species that separated millions of years ago (Lee *et al.* 2021). The relatively stable genomes of laboratory strains of *C. elegans* do not reflect the dynamics of wild nematodes (Denver *et al.* 2009; Meier *et al.* 2014). Given that genomes of wild *C. elegans* strains are quite divergent, understanding nematode genomes and their evolution and variation will require sequencing of many individuals or isolates for each species, either free-living or parasitic.

Ongoing efforts will continue to combine long- and short-read sequence information in an effort to create excellent haploid reference genomes (Doyle *et al.* 2020; Gonzalez de la Rosa *et al.* 2021). Furthermore, the Hi-C technology defines sequences that are physically linked (de Wit and de Laat 2012), thereby facilitating de novo whole-chromosome assembly (Udall *et al.* 2019). The quality of assembled genomes can be assessed based on the fraction of genes present that are highly conserved in a phylum, as determined by programs such as CEGMA or BUSCO (https://parasite.wormbase.org/species.html; Parra *et al.* 2007; Simão *et al.* 2015). Scores of 95% or greater instill confidence in the assembly, whereas scores under 90% suggest a poorly finished genome that should be interpreted with caution. Another measure of genome quality is the N50 statistic, which refers to the size-weighted median of scaffold lengths in a genome assembly. For fully assembled genomes like *C. elegans*, the N50 is 17,493,829 and represents the total length of Chromosome *IV*, which happens to be the smallest contig above the median length. For difficult-to-sequence genomes such as *Caenorhabditis angaria* (https://parasite.wormbase.org/species.html), low BUSCO and N50 numbers indicate substantial uncollapsed haploid genome segments. Genome assemblies are available for hundreds of nematodes (Stevens *et al.* 2020; Susič *et al.* 2020; Teterina *et al.* 2020; Wu *et al.* 2020): https://wormbase.org and https://parasite.wormbase.org/index.html (Kikuchi *et al.* 2017; International Helminth Genomes Consortium 2019). Representative nematode species in Fig. 7 illustrate the quality and diversity of contemporary genome assemblies. Nematodes within the clades considered to be basal (Clades I and II) are currently under-represented.

Nematode genome sizes range 100-fold, from a tiny ∼20 Mb for *Pratylenchus coffeae*, a plant parasite (Burke *et al.* 2015) to 2.5 Gb for *P.* *univalens* (Wang *et al.* 2017), which is close to the 3.2 Gb human genome (International Human Genome Sequencing Consortium 2001). Variation in genome size is due to gene number, intron length, intergenic distance, and repeat content (International Helminth Genomes Consortium 2019). For example, 90% of the 2.5 Gb genome of *P.* *univalens* is made up of 2 short satellite repeats (5-mer = 1.3 Gb and 10-mer = 0.9 Gb; Wang *et al.* 2017). The average nematode genome size ranges from ∼80 to 100 Mb (https://wormbase.org andhttps://parasite.wormbase.org/index.html). In general, genome size does not appear related to nematode lifestyle or habitat (see Table 1, which contains only the highest-quality genome assemblies).

Current gene number estimates range from 6,700 to 34,000 and are typically 15,000 to 25,000. Remarkably, the *P. coffeae* genome contains only slightly more predicted protein-coding genes than the fungi *S. cerevisiae* (5,885) and *Schizosaccharomyces* *pombe* (4,824) (Goffeau *et al.* 1996; Wood *et al.* 2002). Given the caveats noted above, sequencing of *Caenorhabditis* species has shown that genomes range from 65 to 166 Mb, and revealed a surprisingly variable number of protein-coding genes in this genus, ranging from 16,000 to 35,000 (Fierst *et al.* 2015; Yin *et al.* 2018; Kanzaki *et al.* 2018; Stevens *et al.* 2019). Recent phylogenies of *Caenorhabditis* show that the most widely studied species (e.g. *C. elegans*, *C. briggsae*, *C. remanei*) are all part of the *elegans* group of species (Stevens *et al.* 2019). The age of the last common ancestor of *C. elegans* and *C. briggsae* was initially estimated to be ∼80-110 million years (Stein *et al.* 2003), roughly the same distance as between primates and rodents. However, this shrank dramatically, to less than 20 MYA, once fast substitution rates were taken into account (Cutter 2008). Some species pairs are considerably more closely related, with *C. briggsae–C. nigoni* and *C. remanei–Caenorhabditis* *latens* hybrids still retaining partial fertility (Woodruff *et al.* 2010; Dey *et al.* 2012, 2014). Estimates of divergence for more distantly related nematodes (e.g. *C. elegans* and *Pristionchus* ∼200 million years, *C. elegans* and Strongylids ∼380 million years, *C. elegans* and *Ascaris* ∼540 million years, and *C. elegans* and *Trichinella spiralis* >600 million years) have been and remain difficult to accurately estimate (Blaxter 2009).

As nematode genome assemblies improve and additional divergent nematodes are sequenced, our understanding of nematode chromosome organization, synteny, and evolution should be substantially improved, as will the relevance of repetitive sequences to chromosome biology.

### Repetitive and foreign DNA in other nematodes

Transposons are an abundant class of repetitive DNA whose silencing by small RNA pathways was initially worked out in *C. elegans*. Parallel efforts in *Drosophila*, plants and mammals have suggested the evolution of markedly distinct transposon silencing mechanisms, a topic that has more recently been addressed based on analysis of genomes and small RNAs present in diverse nematodes (Sarkies *et al.* 2015). An ancestral eukaryotic genome silencing process targets RNA created from parasites such as transposons by recruiting a processive RNA-dependent RNA polymerase to create dsRNA molecules that are processed by the Dicer nuclease into small RNAs (Castel and Martienssen 2013). Argonaute proteins then interact with the resulting small RNAs and target transcripts from homologous loci in the genome for silencing by recruiting enzymes that promote local deposition of methylation marks on histones or DNA. DNA methylation is present on transposons in the genome of the *T. spiralis* Clade I nematode but has been lost from Clades III–V. Coincidentally, a novel class of nonprocessive RNA-dependent RNA polymerase evolved for Clades III–V, which creates small RNAs de novo in a manner that may amplify and maintain silencing at specific genomic loci (Sarkies *et al.* 2015). Nonprocessive RNA-dependent RNA polymerases can amplify small RNA populations in a manner that creates a persistent form of genome silencing that may substitute for DNA methylation (Sarkies *et al.* 2015), which promotes genomic silencing in vertebrates and plants but has been lost independently from some arthropods, nematodes, and fungi. A second silencing pathway that evolved in metazoans to target transposons employs the Piwi Argonaute protein and a special class of small RNAs termed piRNAs (Almeida *et al.* 2019). Piwi interacts with piRNAs to scan the genome for foreign intruders in Clade V nematodes like *C. elegans* and in mammals and insects, but Piwi and piRNAs have been lost from the other 4 nematode clades, which likely rely on Dicer-mediated small RNAs derived from dsRNA to initiate small RNA silencing of repetitive sequences (Wang *et al.* 2011; Sarkies *et al.* 2015).

*Caenorhabditis* *elegans* lacks 5-methylcytosine, but the presence of this DNA modification in nematodes has been best established for Clade I genera that include *Trichinella* and *Romanomermis* (Gao *et al.* 2012; Schiffer *et al.* 2013). Loss of 5-methylcytosine in nematode evolution occurs in concert with loss of the DNA alkylation enzyme ALKB2, which detoxifies the 3-methyl cytosine byproduct of 5-methylcytosine (Rošić *et al.* 2018). Moreover, reports of N6-methyldeoxyadenine in *C. elegans* suggest that this DNA modification, which occurs in bacteria, archaea, protists, and fungi, may be present in nematodes (Greer *et al.* 2015; Ma *et al.* 2019; O’Brown *et al.* 2019). The variable presence of at least 2 types of DNA methylation in diverse kingdoms of life suggests mechanisms that have been widely lost and/or spread during evolution, possibly originating on independent occasions from prokaryotic restriction modification systems in a manner that may have broadly impacted eukaryotes.

During the evolution of *Caenorhabditis* nematodes, self-fertile androdioecious species arose several times from male to female outcrossing (gonochoristic) ancestors. Genome sizes of self-fertile species became consistently smaller than outcrossers. Large-scale changes to genome size were not primarily orchestrated by changes to transposon or repeat copy number. Instead, changes to gene number that might promote adaptation to self-fertilization are thought to be drivers of genomic change (Fierst *et al.* 2015). In contrast, enormous tandem repeat expansions are partially responsible for large increases in genome size in some parasitic nematodes like *P.* *univalens* (Wang *et al.* 2017). Tandem repeats are the simplest class of repetitive DNA and can be composed of short microsatellite (1–6 nt) or longer minisatellite (7–180 nt) repeat units. The total length of tandem repeat tracts can reach 50 kb in the *C. elegans* genome and much greater lengths (200 Kb to 1 Gb) in other nematodes. The presence of large numbers of tandem satellites in nematodes has been suspected to play a role in chromosome structure and function, for example by promoting correct recombination (Subirana and Messeguer 2010; Subirana *et al.* 2015). Although tandem repeats are noncoding segments of the genome, their expression in mammals can occur in response to stress and the expansion of tandem repeat tracts that occur in either coding and noncoding mRNA segments is responsible for a number of inherited disorders (Jolly *et al.* 2004; Rizzi *et al.* 2004; Zhang and Ashizawa 2017). The sequences of longer tandem repeat tracts can be heterogeneous such that polymorphisms occur for many repeat units within a tandem repeat tract, which can make quantifying tandem repeat tract RNA expression from next-generation sequencing challenging. Some tandem repeats of the genome can encode important cellular products such as ribosomal or histone RNAs and in some nematodes, the spliced leader RNA involved in trans-splicing. However, histone loci are dispersed in the *C. elegans* genome with only several histone repeats per locus (Boeck *et al.* 2016), in contrast to a single histone locus in *Drosophila* with ∼100 repeats (McKay *et al.* 2015).

Studies of plant-parasitic nematode genomes have revealed that horizontal transfer of genes from soil bacteria or fungi may be a common mechanism that promotes metabolic innovation in nematodes (Eves-van den Akker *et al.* 2016; Kikuchi *et al.* 2017). Another common feature of nematode chromosomes is large or small tracts of DNA transmitted from intracellular bacteria like *Wolbachia* that infects several parasitic nematodes (Dunning Hotopp *et al.* 2007; Slatko *et al.* 2014). This may reflect a process that resembles the nearly complete transfer of genomic DNA from the endosymbionts mitochondria or chloroplasts into the nuclear genomes of eukaryotes. *Wolbachia* is present in the majority of insect species and may confer a selective advantage to species it infects, such as resistance to viruses (Hedges *et al.* 2008). It has become so important for some filarial nematode life cycles, in part by providing metabolites that the nematodes can no longer synthesize, that antibiotics targeting *Wolbachia* can cure these nematode infections (Landmann 2019).

### Chromosome organization in other nematodes

The general organization of the autosomes and X chromosome of *C. elegans* is conserved in some other *Caenorhabditis* species and in the more distantly related Clade V nematode *P.* *pacificus* (Rödelsperger *et al.* 2017; Prabh *et al.* 2018; Werner *et al.* 2018). However, in another Clade V nematode, *Haemonchus contortus* (a parasite of ruminants), the gene and repeat distribution along the length of autosomes are more uniform than in *C. elegans* (Doyle *et al.* 2020). Similarly, a relatively uniform distribution of genes and repetitive sequences along the autosomes is present in some nematodes from Clades III (*A.* *suum*) and IV (*Strongyloides ratti*; Hunt *et al.* 2016; Wang *et al.* 2017). Additional nematode chromosome assemblies and recombination analyses, particularly in more basal clades, will provide further insight into nematode chromosomes.

Synteny is the similar ordering of DNA sequences among distinct chromosomes or regions. In the case of macrosynteny, the syntenic region is very large, up to an entire chromosome. A remarkable degree of chromosome-level synteny is evident among the members of Clade V, as revealed by the ordering of best-matching protein orthologs in the fully sequenced genomes of *C. elegans* and its distant relatives *P. pacificus* (Lee *et al.* 2003; Rödelsperger *et al.* 2017) and *Auanema rhodensis* (Tandonnet *et al.* 2019), as well as its closer relatives *C. briggsae* and *C. inopinata* (Hillier *et al.* 2007; Kanzaki *et al.* 2018). Comparisons of these species revealed that hundreds of intrachromosomal translocations and inversions have occurred within each chromosome and that individual genes can move to different chromosomes. However, there are no large rearrangements involving different chromosomes observed among *Caenorhabditis* species, and only a single large rearrangement in *Caenorhabditis* relative to *Pristionchus*. Repression of interchromosomal rearrangements has also been noted within Clade IV (Hunt *et al.* 2016), so this may be a general theme in nematode chromosome biology. The absence of interchromosomal translocations after the *elegans*/*briggsae* split is highly significant when compared to the large number of interchromosomal translocations observed between mice and humans or within the primate lineage (Hillier *et al.* 2007). It is perhaps even more surprising that synteny and chromosome number have been so well-maintained in the *Caenorhabditis* genus, since translocations or chromosome fusions should create stable new holocentric chromosomes rather than unstable acentric or dicentric chromosomes that can occur in monocentric organisms (see below).

A recent study (Tandonnet *et al.* 2019) used this extensive macrosynteny to analyze current karyotypes of a wide sampling of Clade V nematodes in terms of an ancestral set of 6 or 7 linkage groups termed Nigon elements. Why ancestral linkage groups in Clade V have been so well-preserved over tens of millions of years is an open question. Possible explanations discussed by (Hillier *et al.* 2007) include: (1) a large effective population size, which would reduce the probability of translocations becoming fixed; (2) strong selection for maintaining the arm/center distinction of chromosomes, which may be disrupted by translocations; and (3) the possible existence of sequence motifs imparting chromosome-specific identity used in critical processes such as homologous pairing in meiosis. Possibly, similar considerations may explain the striking constancy in overall karyotype in Clade V members, most of whom have a chromosome number of 6 (Mitreva *et al.* 2005). It appears more plausible that postrepair selection, rather than strong innate bias against interchromosomal repair, might account for the constancy of karyotype, since interchromosomal translocations can be made relatively easily in *C. elegans* (e.g. translocation heterozygote balancer chromosomes) and other species (Edgley *et al.* 2006). As more whole nematode genomes are sequenced and assembled, it will be informative to track variations in macrosynteny and the depth at which it breaks down, and whether there are any common breakpoints in intrachromosomal rearrangements between different species. If the remarkable preservation of macrosynteny is due to either of the possibilities (2) or (3) above and is not just a result of a large effective population size, this would reflect a previously underappreciated level of regulation across whole chromosomes. Moreover, genome sequence from other clades would shed light on whether this preservation of macrosynteny is a nematode-wide phenomenon or something specific to Clade V.

### Telomeres and telomerase in other nematodes

Most nematode chromosomes are capped with (TTAGGC)_n_ telomeric repeat, but a known exception is *D.* *pachys* (Fradin *et al.* 2017), which has a single haploid chromosome. Analysis of the *Diploscapter* genome revealed that it lacks (TTAGGC)_n_ telomeric repeat tracts (Fradin *et al.* 2017). Further, genome and proteome analyses failed to identify genes encoding the TRT-1 telomerase reverse transcriptase and POT-1 and POT-2 single-stranded telomere-binding proteins (Fig. 5c), whereas the *lmn-1* lamin gene that is adjacent to *trt-1* in *C. elegans* is clearly present. *Diploscapter* reproduces in an asexual manner in the absence of hallmarks of meiotic recombination (Kiontke and Fitch 2005), which might allow for intergenerational transfer of a circular chromosome, which is known to be permissive for mitosis but toxic for meiosis in *S. pombe* (Baumann and Cech 2001). However, *Diploscapter* chromosomes cytologically appear linear, indicating that canonical telomeres in this nematode could have been replaced by an alternative system, such as the non-LTR retrotransposons that cap the telomeres of *Drosophila* (Mason *et al.* 2008).

The (TTAGGC)_n_ telomere repeats at most subtelomeres of the *C. elegans* genome begin with the nucleotides TTA or CTT (Wicky *et al.* 1996). Consistent with this, a de novo telomere created in the *C. elegans* germline, *me8*, is a truncation of the *X* chromosome where the site of de novo telomere addition begins with the sequence TTA (Wicky *et al.* 1996). In contrast, the telomere–subtelomere junctions of the germline chromosomes of *Ascaris* exhibit no sequence preference (Wang *et al.* 2020). Furthermore, sequence analysis of telomeres created de novo at the ends of broken germline chromosomes during somatic development of the parasitic nematodes *A.* *suum* and *P.* *univalens* revealed that a single nucleotide of telomere sequence homology is an optimal target of telomerase in these species (Jentsch *et al.* 2002; Wang *et al.* 2017). Overall, these results imply that *Ascaris* and *Parascaris* telomerase is promiscuous in both germline and somatic cells, whereas *C. elegans* telomerase may have greater target specificity. That said, the limited homology observed at double-strand breaks targeted by telomerase in nematodes is consistent with the need for only a few nucleotides of homology for oligonucleotides that are effective substrates of ciliate or human telomerase in vitro (Collins 1999).

### Perspectives on nematode holocentric chromosomes

In addition to nematodes, holocentric chromosomes are also present in some insects, arachnids, and plants (Cuacos *et al.* 2015; Marques and Pedrosa-Harand 2016). Why do organisms have holocentric chromosomes? Holocentricity allows for creation of stable chromosome fusions and fissions, which in principle allows for highly flexible chromosome evolution. Yet, paradoxically, nematode chromosomes have unusually stable karyotypes. A recent study examining whether holocentric chromosomes constitute an evolutionary advantage in terms of diversification and species richness concluded that the potential benefits or adaptation of monocentromeres vs holocentromeres appear unrelated to specific chromosome behaviors (Márquez-Corro *et al.* 2018). Although karyotyping studies on most nematodes suggest their chromosomes are holocentric, the Clade I (Dorylaimia, Trichinellida) parasitic nematodes *Trichinella* and *Trichuris* exhibit chromosome constrictions and thus may be monocentric (Spakulova *et al.* 1994). Therefore, monocentric and holocentric chromosomes may each possess different evolutionary advantages that may contribute to the success of some nematode species (Márquez-Corro *et al.* 2018). Studies suggest that the centromeres of monocentric chromosomes play a role in genome architecture and 3D nuclear organization (Muller *et al.* 2019). Analysis of this architecture and organization for holocentric chromosomes might provide additional insight into how centromeres impact genome architecture as well as their contribution to chromosome structure and function and gene expression.

*Caenorhabditis* *elegans* kinetochores are dependent on CENP-A during mitosis but not in meiosis (Monen *et al.* 2005). In *C. elegans* oocytes, chromosome congression and alignment involves lateral attachment of microtubule bundles to chromosomes (Wignall and Villeneuve 2009; Muscat *et al.* 2015), while segregation appears to use an as-yet unresolved combination of both pushing forces from the central spindle as well as kinetochore-dependent pulling (Dumont *et al.* 2010; Laband *et al.* 2017; Yu *et al.* 2019; Danlasky *et al.* 2020; reviewed in Taylor and Pelisch 2020). In *C. elegans* males, the bivalent end facing the poles interacts with microtubules that directly insert into chromatin (Albertson and Thomson 1993; Monen *et al.* 2005). Similarly, microtubules in *Parascaris* do not use kinetochores during male meiosis, but rather insert directly into the tips of heterochromatic chromosome arms; whereas in female meiosis a well-defined kinetochore plate appears to extend along the length of oocyte chromosomes (Goldstein 1977, 1978; Goday and Pimpinelli 1989; Pimpinelli and Goday 1989). Overall, studies of nematode chromosome segregation indicate that the dispersed holocentromere that is present in mitosis can be reprogrammed during meiosis and even during embryonic development. The mechanism that regulates microtubule attachment near meiotic chromosome termini is an intriguing question.

While holocentric and monocentric chromosomes carry out mitotic segregation in a very similar manner, segregation during meiosis requires special consideration. In meiosis, paired homologous chromosomes, consisting of 4 individual chromatids, undergo 2 rounds of segregation. The 4 chromatids are linked to each other by chiasmata (exchanges of DNA continuity caused by crossover recombination) and held together by sister chromosome cohesion. Cohesion must be removed in 2 discrete steps, in meiosis I and II, to create gametes containing a single chromatid for each chromosome. The 2-step loss of cohesion enables chromatid pairs that have segregated away from each other in the first meiotic division to remain linked until the second division. In the majority of eukaryotes investigated, these 2 discrete cohesion loss events are controlled by centromere-resident proteins, such as Shugoshin (Kitajima *et al.* 2004, 2006), which recruit factors that protect centromeric cohesion during meiosis I. Regulated loss of this protection in meiosis II allows the second division to take place.

Holocentric chromosomes do not possess defined centromeric and noncentromeric domains that can compartmentalize the early and late loss of cohesion. Nevertheless, since the endpoint of meiosis (creation of haploid cells from initial diploid cells) is the same in holocentric organisms, a controlled 2-step loss of cohesion must still be carried out. Studies in *C. elegans* oocyte meiosis have revealed an alternative mechanism that establishes 2 functionally distinct domains of unequal size on each chromosome and ensures that sister cohesion is lost first from one domain, then the other. The unequal size of these domains derives from the biased off-center position of the single meiotic crossover (Albertson and Thomson 1993; Rockman and Kruglyak 2009; Yokoo *et al.* 2012) that divides each pair of homologs into a short arm and a long arm. Strikingly, the domain in which cohesion is lost first is always the short arm. In early prophase, these domains differentially recruit several factors that during the meiosis I division orchestrate cohesion loss on the short arm and cohesion protection on the long arm (de Carvalho *et al.* 2008; Martinez-Perez *et al.* 2008; Tzur *et al.* 2012; Ferrandiz *et al.* 2018; Sato-Carlton *et al.* 2018).

**Fig. 8.**
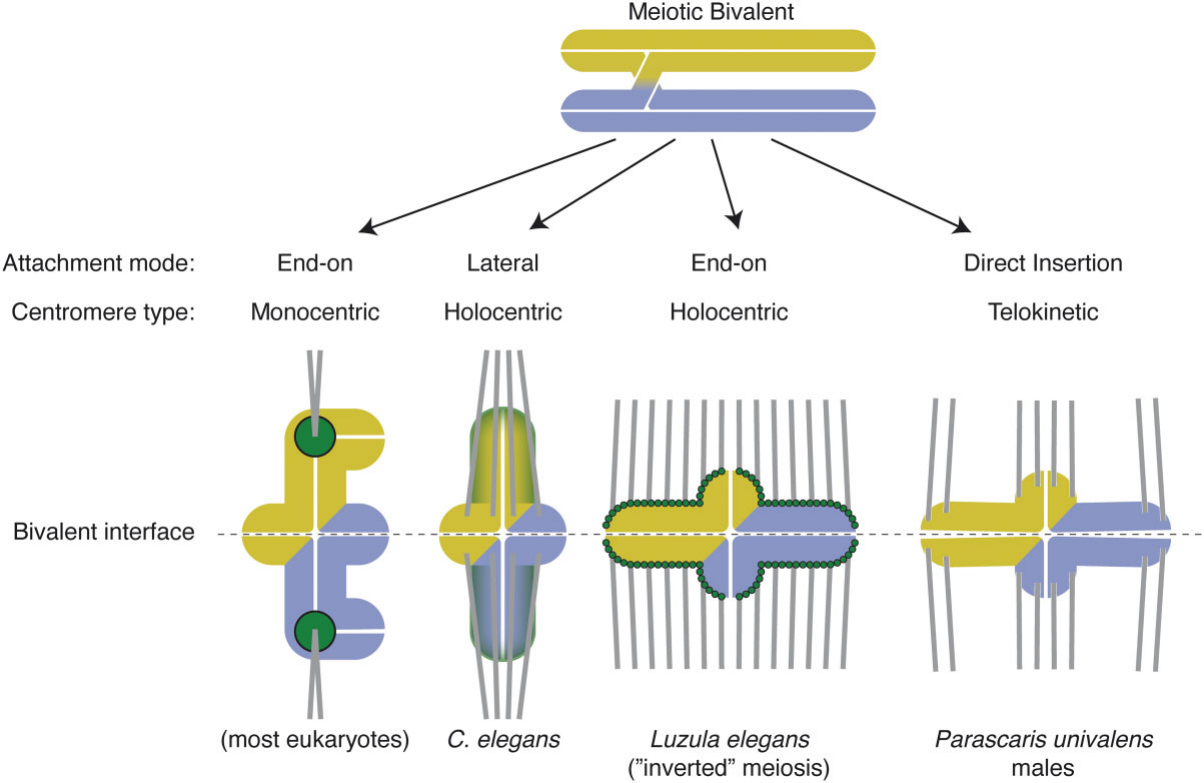
Comparison of chromosome segregation for different types of centromeres and microtubule attachment modes. In all cases, sister chromatid cohesion must be removed at the bivalent interface, and protected on at least some part of the remaining linked chromatid pairs, for correct 2-step segregation. The diagram for telokinetic segregation is speculative, based on Figure 3a of Goday and Pimpinelli (1989).

Since crossovers can occur anywhere on the chromosome, short and long arm identity must be facultatively established for each chromosome in each meiotic cell, implying the existence of a mechanism that somehow senses the distance from the crossover to the nearest chromosome end. The higher rate of crossing-over in the terminal regions of chromosomes (Barnes *et al.* 1995; Rockman and Kruglyak 2009) may enhance the robustness of this mechanism, since the length difference between the short and long arms is usually substantial. However, as pointed out in (Albertson *et al.* 2011), although crossovers are not biased toward chromosome ends in the *rec-1* mutant, this mutant has completely normal meiotic chromosome segregation; therefore, a large difference in arm length is not strictly necessary for successful meiosis I disjunction. Furthermore, even in *syp-1* mutant strains that fail to achieve arm-specific localization of proteins such as HTP-1 and LAB-1, correct disjunction occurs in more than half of oocytes (Sato-Carlton *et al.* 2018; Garcia-Muse *et al.* 2019), hinting that multiple mechanisms redundantly ensure a 2-step loss of cohesion in meiosis. A simple possibility for such a mechanism could be the geometrical tendency of lateral attachment of microtubules to orient chromosomes along their long axis (since more microtubules can attach to long arms than to short arms), with concurrent action of separase, the protein that degrades cohesin (Siomos *et al.* 2001), at the bivalent interface. This would be sufficient to promote proper chromosome disjunction for any type of chromosome (Fig. 8).

The loss of monocentrism in holocentric nematodes, and by extension other holocentric species, must have coincided with innovations for achieving 2-step meiotic chromosome segregation. The peculiar system of chromosomal domain specification by the placement of crossovers has been extensively investigated in *C. elegans*, but how well this system is conserved in other Rhabditids or in more distant nematode taxa is not known. One example of a nematode using a different system is the first meiotic division in male *P.* *univalens*; as mentioned above, each homolog is apparently pulled by both of its heterochromatic ends during anaphase I (Goday and Pimpinelli 1989). This form of division has been termed “telokinetic”; however, a detailed picture of the chromosome conformation at this stage remains elusive. Outside of nematodes, several organisms with holocentric chromosomes such as the plant *Luzula elegans* are known to undergo an “inverted” meiosis, in which the first meiotic division is equational, and the second reductional (Heckmann *et al.* 2014). In the case of chiasmate holocentric meiosis, however, since the distinction between sister and homolog disjunction is a matter of degree, inverted meiosis may be seen as a 90° rotated variation on *C. elegans* holocentric meiosis, with end-on rather than lateral microtubule attachment (Fig. 8).

Another remarkable aspect of *C. elegans* meiotic chromosome segregation is biased segregation of chromosomes of unequal size during spermatogenesis. It has been found that heterozygous insertions or free duplications have a size-dependent tendency to segregate away from the single male X chromosome, a phenomenon termed skew (Wang *et al.* 2010). As a result of skew, in the presence of unequal-sized chromosomes, the hermaphrodite offspring of males tend to receive the shorter chromosome, while the male offspring of males tend to receive the longer chromosome. While the mechanism of this skewed inheritance is still not understood, it appears to be a major factor shaping genome evolution in all species of the genus *Caenorhabditis* (Le *et al.* 2017), likely explaining the observation that the androdioecious species all have smaller genomes than their gonochoristic relatives (Yin *et al*. 2018).

### Programmed DNA elimination in nematodes

**Fig. 9.**
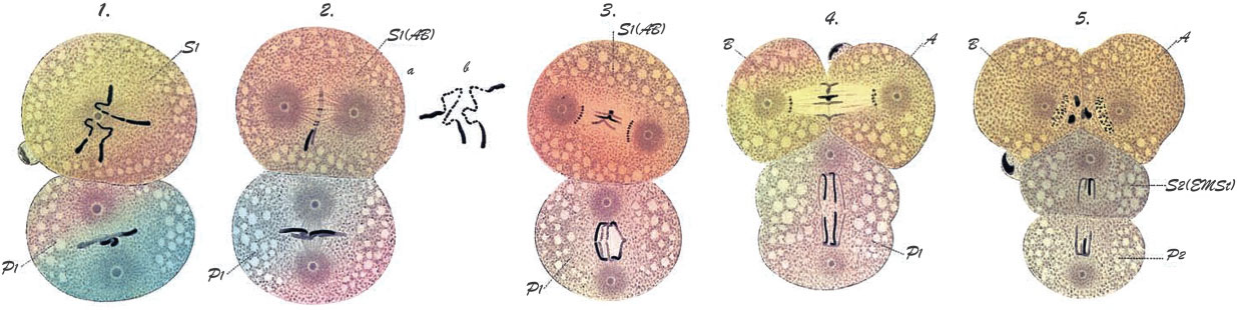
Programmed DNA elimination in early development of *Parascaris univalens*. (1) Two-cell stage (St, somatic cell; P, germline cell). (2a) Two-cell stage with the somatic cell in metaphase of a mitotic division. (2b) Higher-resolution illustration of diploid chromosomes illustrating the thick heterochromatic arms that undergo DNA elimination and the bead-like nature of the central region of the chromosomes. (3) Anaphase of somatic cell division illustrating smaller and multiple chromosomes being segregated with large chromosome fragments remaining at the metaphase plate. The germline cell has entered into mitosis. (4) Telophase and cytokinesis of the somatic cell and anaphase of the germ cell. (5) Four-cell stage illustrating the 2 somatic cells (A and B). On formation of the nuclear membrane, there will be 29 haploid somatic chromosomes and 6 or 12 X chromosomes, male or female, respectively. The large fragments of DNA that will be lost are relegated to the cytoplasm and eventually degraded. The germline cell has undergone division into a somatic cell (EmSt) and a germline cell (P2). See text and Figs. 10 and 11 for additional description of DNA elimination [from Boveri (1899) as modified by Satzinger (2008)]

Genome integrity and its maintenance are essential to an organism, since chromosome loss in whole or part may remove essential genes. In most organisms, therefore, genetic information derived from the zygotic nucleus is thought to persist virtually unchanged in all cells during the entire lifespan. However, there are examples where developmentally regulated DNA loss is an integral process in the biology of organisms. Programmed DNA elimination, where there is conserved, regulated, and reproducible loss of specific DNA sequences during the life cycle of an organism, was first observed in a nematode in 1887 by Boveri (1887). Boveri observed in early embryo development of the horse parasite *P.* *univalens* (then called *A.* *megalocephala*) that in early somatic cells the large chromosomes underwent numerous chromosomal breaks (Fig. 9). The large heterochromatic arms of the chromosomes were not segregated at anaphase of mitosis, but instead were localized to the cytoplasm following cell division and eventually were degraded and lost. The remaining retained chromosome fragments consisted of 29 haploid autosomes and either 6 or 12 X chromosomes in the male or female, respectively (Niedermaier and Moritz 2000). Chromosome breaks and subsequent retention and loss of chromosome fragments occurred only in somatic cells, resulting in distinct somatic and germline genomes, with a reduced genome in somatic cell lineages. Programmed DNA elimination or programmed genome rearrangements have subsequently been identified in a broad range of organisms including ciliates and some insects, mites, copepod crustaceans, lampreys/hagfish, songbirds, and marsupials (Wang and Davis 2014).

**Fig. 10.**
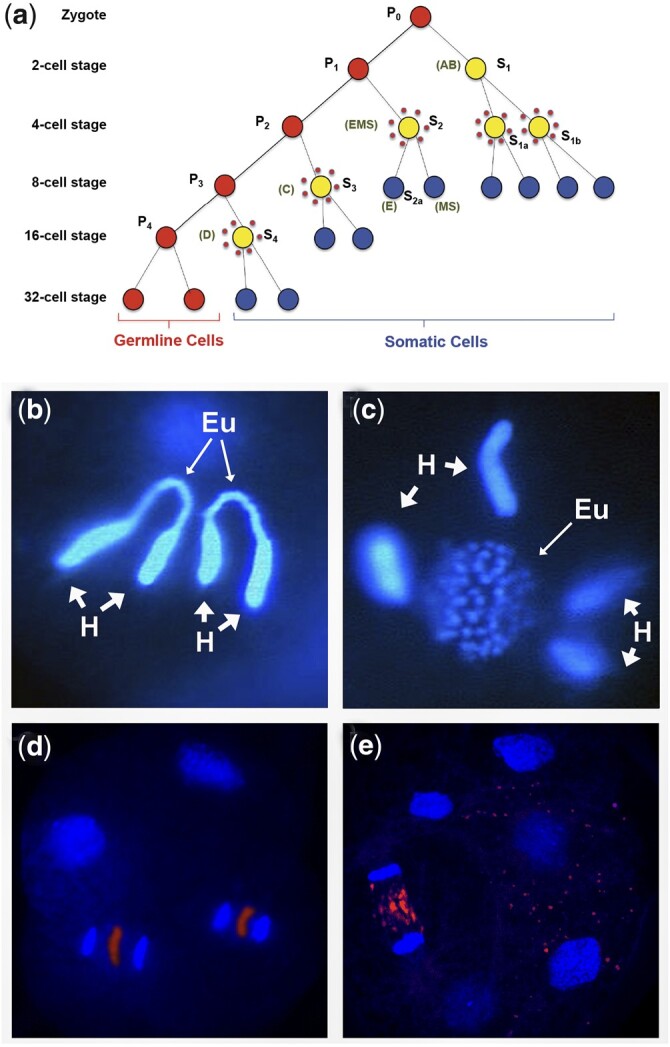
Ascaris early embryo development, cell lineage, and DNA elimination. a) Primordial germ cells (P) are in red, cells undergoing DNA elimination are represented by yellow-filled circles surrounded by red dots (representing the eliminated DNA), and blue cells (S) are precursor somatic cells and lineages. The primordial germ cell numbers correspond to their division state. P0 is the zygote, whereas P1 through P4 represent the primordial germ cell derived from each subsequent cleavage of the germ cells as illustrated. S1–S4 cells are successive precursor somatic cells derived from each division of a germ cell. Adapted from original Boveri presentation [see Streit *et al.* (2016)]. b and c) *Parascaris univalens.* b) Two-cell embryo showing one cell with the single pair of germline chromosomes. The big arrows indicate the heterochromatic arms of the chromosomes (H) and the small arrows point to the euchromatic regions of the genome (Eu). c) Somatic cell undergoing programmed DNA elimination from a 2-cell embryo. The retained portions of the germline chromosomes (Eu) are fragmented into ∼2*n* = 70 chromosomes. The heterochromatic arms that will be eliminated (H, big arrows) remain visible. d, e) *Ascaris suum*. d) Four-cell embryo with 2 cells undergoing DNA elimination (65 h) and e) 6-cell embryo with one cell undergoing DNA elimination (∼80 h). Note that DNA to be eliminated is present as fragments (artificially colored in red) between segregating chromosomes in early anaphase. DNA fragments derived from a previous elimination event can still be seen in the cytoplasm of cells in (e). Modified from Streit *et al.* (2016).

Current methods for identification of DNA elimination include cytological analysis of chromosomes during mitosis (see Fig. 10, b–e), which may be insensitive if only small portions of a genome are eliminated, and comparisons of genome sequences from germline and somatic cells. Few comprehensive studies like these have been done on nematodes. To date, programmed DNA elimination in nematodes has been described in the 3 Rhabditida suborders: Spirurina (Ascaridomorpha, including *Parascaris, Ascaris*, and others), Tylenchina (Panagrolaimomorpha, *Strongyloides* spp.; Streit *et al.* 2016), and in the Rhabditina (*Oscheius*; Gonzalez de la Rosa *et al.* 2021). It does not appear to occur in *C. elegans* (Emmons *et al.* 1979; Wang *et al.* 2017). Recent telomere to telomere genome sequencing of *Ascaris* indicates that, in addition to the previously described intrachromosomal breaks, all chromosome ends undergo chromosome remodeling through subtelomeric DNA breaks, loss of distal sequences, and healing of the ends by telomere addition (Wang *et al.* 2020). Recent comprehensive sequencing of *Oscheius tipulae* (Rhabditina; Clade V) also identified chromosome end remodeling by DNA elimination through subtelomeric DNA elimination (Gonzalez de la Rosa *et al.* 2021). Programmed DNA elimination has recently been identified in *Caenorhabditis mondelphis* (Gonzalez de la Rosa PM, Stevens L, Blaxter ML, personal communication). As more telomere-to-telomere and comprehensive genome assemblies are generated, DNA elimination may be found to occur in more nematodes.

Two types of programmed DNA elimination are observed in nematodes: (1) double-strand breaks in chromosomes with some portions of chromosomes retained and other portions eliminated and (2) elimination of entire chromosomes. The first form has been studied more and is best understood in *Ascaris* (Wang *et al.* 2012, 2017, 2020), an intestinal parasite of humans and pigs. DNA elimination occurs in 5 distinct presomatic cells during the 4–16 cell stage of early development (Fig. 10a). DNA elimination in *Ascaris* somatic cells includes 72 DNA breaks and results in an increase in chromosome number (24–36), and loss of 18% of the genome (55 Mb). No chromosome fusions or rearrangements have been observed, and the broken ends of the retained chromosomes are healed by the addition of telomeres. All chromosome ends undergo remodeling during *Ascaris* DNA elimination: the chromosomes undergo subtelomeric breaks, loss of distal sequences including the telomeres, and de novo telomere healing of the chromosome ends (Wang *et al.* 2020). How this may affect gene expression due to “position effects” and what impact this has on chromosome architecture or 3D organization remains to be determined. Analysis of the eliminated DNA indicates that 70% is repetitive DNA (35 Mb DNA), primarily a 120 bp satellite repeat. Strikingly, 1,000 genes (∼5% of all genes, 20 Mb DNA) are also eliminated, and these genes are expressed primarily in the germline or very early in development. The chromosome breaks and eliminated DNA are the same in all 5 presomatic cells (Fig. 10a, yellow cells with red dots), and the DNA elimination and formation of new chromosomes occur with high fidelity among worms including both males and females.

Additional comparative analysis of DNA elimination in other related ascarids (*P.* *univalens*, a parasite of horses, and *Toxocara canis*, a parasite of dogs whose larvae can infect humans) indicates that the process is also highly regulated and occurs with high fidelity as observed in *Ascaris* (Wang *et al.* 2017). The majority of eliminated DNA is repetitive sequence that varies in each genus (*T.* *canis *=* *49 bp satellite repeat; *P.* *univalens *=* *5 and 10 bp satellites). Almost 90% of the germline genome is eliminated to form the somatic genome in *P.* *univalens*. Large numbers of genes (1,000–2,000, 5–10% of the genome) are also eliminated in these 2 genera. As in *Ascaris*, the eliminated genes are primarily expressed in the germline and early embryo. Thirty-five percent of the eliminated genes are shared among the 3 genera (*Ascaris*, *Parascaris*, and *Toxocara*), perhaps representing the key genes eliminated, and these genes are primarily expressed during spermatogenesis. Overall, these data suggest that one function of programmed DNA elimination in nematodes serves to permanently silence germline expressed genes in somatic cells.

The location of the chromosome breaks, healing of the broken ends by telomeres, and selection of the DNA eliminated all occur reproducibly and with high fidelity within all 3 genera. Among the genera, however, the location of the breaks and the eliminated DNA are different. While the location of the chromosome DNA breaks occurs with high fidelity at the chromosome level, the breaks do not occur at a precise DNA sequence, but instead occur within a 3–6 kb region known as a chromosome break region. Analysis of these break regions has not identified any characteristic sequence or structural motifs, and no specific epigenetic marks or small RNAs appear associated with these breaks. Interestingly, in *Ascaris*, chromosome break regions become more accessible (based on ATAC-seq) just prior to and during DNA elimination (Wang *et al.* 2017, 2020). The identification of break sites has been based on sites of de novo telomere addition, and the loss of sequences distal to the site in the somatic genome when compared to the germline genome (Wang *et al.* 2012, 2017, 2020). This identifies where telomere healing occurs (which may occur at any nucleotide within the chromosome break region indicating no nucleotide requirements for telomere addition). Preliminary experiments using END-seq (Canela *et al.* 2016) to identify the break sites suggest that they are also heterogeneous within the chromosomal break regions (Wang J and Davis RE, unpublished results). It is currently not known if the site of the breaks is where telomeres are added, but the identification of breaks without telomere healing suggests the 2 processes are temporally separated. The mechanism for how a break is generated is unknown. However, the lack of sequence specificity, the openness of chromatin, and heterogeneity of break location within the 3–6 kb region suggest that the breaks are a consequence of a nonsequence-specific process.

**Fig. 11.**
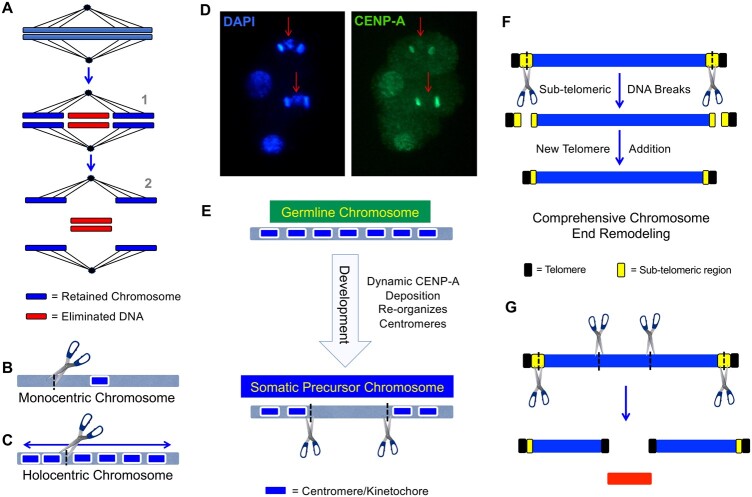
Models for *Ascaris* DNA elimination and mechanism for the loss of chromosomal regions from holocentric chromosomes. a) Model of internal DNA elimination (Wang *et al.* 2017). Following alignment, chromosomes in somatic cells undergo chromosome breaks producing fragments of chromosomes. Chromosome fragments that are retained (blue) have centromeric sites for microtubule attachment to facilitate chromosome segregation, whereas chromosomal fragments that will be eliminated (red) remain at the metaphase plate, are not segregated, and are lost. b) Monocentric chromosomes have a single centromere (blue box) where spindle microtubule attachment occurs. Fragmentation of a monocentric chromosome would likely lead to a loss of acentric chromosomal regions during chromosome segregation. c) Holocentric chromosomes have multiple centromeric regions distributed along the chromosome length that serve as sites for microtubule attachment. This distribution of microtubule attachment sites would predict no loss of chromosomal fragments following chromosome breakage during DNA elimination. d) CENP-A is reduced in genomic regions that are not segregated to daughter nuclei in a DNA elimination mitosis. These genomic regions remain at the metaphase plate during anaphase and will be lost during DNA elimination. Immunostaining of CENP-A in a 4-cell *Ascaris* embryo with 2 cells undergoing DNA elimination mitoses (anaphase) indicates the DNA to be eliminated (red arrows) has much less CENP-A than the DNA that will be segregated and retained. e) CENP-A and centromeres/kinetochores in germline *Ascaris* chromosomes are distributed along the length of the chromosomes. During development, CENP-A deposition is reduced on regions of chromosomes that will be lost during DNA elimination. Thus, dynamic CENP-A deposition defines and regulates which portions of chromosomes will be retained and which will be lost during DNA elimination. f) Model of chromosome end remodeling. All chromosome ends undergo subtelomeric DNA breaks. The broken ends of the chromosomes are healed by telomere addition. g) Integrated model illustrating internal chromosome breaks leading to 2 new chromosomes and chromosome end remodeling. Modified from Streit *et al.* (2016).

As described earlier, *C. elegans* has holocentric chromosomes. If *Ascaris* had similar holocentric chromosomes, it would be unlikely that any chromosome fragments would be lost during DNA elimination as centromeres/kinetochores are distributed along the length of the chromosomes. Using antibodies developed against *Ascaris* CENP-A with immunostaining and genome-wide ChIP-seq experiments, it was found that *Ascaris* chromosomes in the mitotic germline (male and female reproductive system) are fully holocentric as observed in *C. elegans* (Kang *et al.* 2016). However, during late gametogenesis and in the early embryo, regions of chromosomes that will be lost as a consequence of DNA elimination exhibit reduced CENP-A and kinetochore components. Thus, the process of DNA elimination involves both specific chromosome breaks and dynamic changes in CENP-A/kinetochores in regions that define which chromosome fragments will be kept (segregated during anaphase) and which will be eliminated (those that lack CENP-A/kinetochores and are not segregated; Kang *et al.* 2016) (Fig. 11). The mechanism for how these changes in CENP-A localization occur is unknown. As shown in Fig. 11, one model is that the chromosome breaks would most likely occur at the metaphase plate, since if the breaks occurred earlier in the cell cycle, chromosome fragments lacking centromeres/kinetochores might not be able to congress to the metaphase plate. Strikingly, preliminary END-seq data (Wang J and Davis RE, unpublished results) and ultrastructural analysis (Wang *et al.* 2020) suggest that chromosome breaks may occur prior to metaphase and congression of the chromosome fragments to the metaphase plate during DNA elimination might involve the contribution of polar ejection forces involving dynamic instability of pole-initiated microtubules, plus-end directed chromokinesins, interpolar microtubules, and/or scaffolding or tethering proteins within the spindle. DNA for elimination is sequestered into micronuclei at telophase during mitosis. The chromatin in the micronuclei undergoes loss of active histone marks, and the eliminated DNA appears to be degraded through autophagy (Wang *et al.* 2020).

In ciliates that undergo programmed DNA rearrangements, which also includes DNA elimination, small RNAs, and in some cases, long RNAs play key roles in identifying sequences that will be kept vs those that will be lost from the micronucleus (germline genome) in forming the macronucleus (somatic genome; Betermier and Duharcourt 2014; Yerlici and Landweber 2014; Noto and Mochizuki 2017). Like *C. elegan*s (Grishok 2013; Almeida *et al.* 2019), *Ascaris* exhibits a complex set of small RNAs including miRNAs, 22G-RNAs, and 26G-RNAs, but lacks piRNAs (Wang *et al.* 2011; Zagoskin *et al.* 2021). piRNAs are also absent in many other nematodes (Sarkies *et al.* 2015). Extensive analysis of small RNAs prior to and during DNA elimination in *Ascaris* has not identified small RNAs that target the chromosome break regions, localization of CENP-A, or the retained compared to eliminated DNA sequences (Wang *et al.* 2011; Zagoskin M, Wang J and Davis RE, unpublished results). However, a WAGO Argonaute (similar to *C. elegans* WAGO-1) has been observed using immunostaining to be only on retained chromosomes while another (similar to *C. elegans* C04F12.1/VSRA-1) is enriched on chromosome fragments that will be eliminated during a DNA elimination mitosis (Wang *et al.* 2011; Zagoskin M, Wang J and Davis RE, unpublished results). Argonaute proteins can function by altering chromatin state and impact gene expression (Almeida *et al.* 2019; Shuaib *et al.* 2019; Weiser and Kim 2019). However, the role of these Argonautes and their associated small RNAs in DNA elimination remains to be determined.

The recent telomere-to-telomere assembly of the free-living nematode *O.* *tipulae* revealed that the subtelomeric regions of all 6 chromosomes undergo chromosome end remodeling through DNA elimination (Gonzalez de la Rosa *et al.* 2021). The amount of eliminated DNA is relatively small, ranging from 4 to 133 Kb in length, and totaling 349 Kb (only ∼0.5% of the genome). In contrast to DNA elimination in ascarids, the site at which the end of the germline chromosome is broken and a telomeric repeat added appears to be highly precise and occurs within specific sequences in *O. tipulae* (Gonzalez de la Rosa *et al.* 2021). Sequencing of several other *Oscheius* species has revealed that DNA elimination is widespread in the genus with a short palindromic motif present where the DNA breaks and telomere addition occurs (Gonzalez de la Rosa PM, Stevens L, Blaxter ML, personal communication). Some *Oscheius* species also undergo internal chromosome DNA elimination, leading to chromosome breakage and thus karyotypic differences between the germline and somatic genomes (Gonzalez de la Rosa PM, Stevens L, Blaxter ML, personal communication). While DNA elimination is absent in *C. elegans*, it has recently been discovered in an early diverging species of *Caenorhabditis*, *C. mondelphis*, raising the interesting possibility that programmed DNA elimination was the ancestral state in *Caenorhabditis* and has since been lost in some species (Gonzalez de la Rosa PM, Stevens L, Blaxter ML, personal communication).

**Fig. 12.**
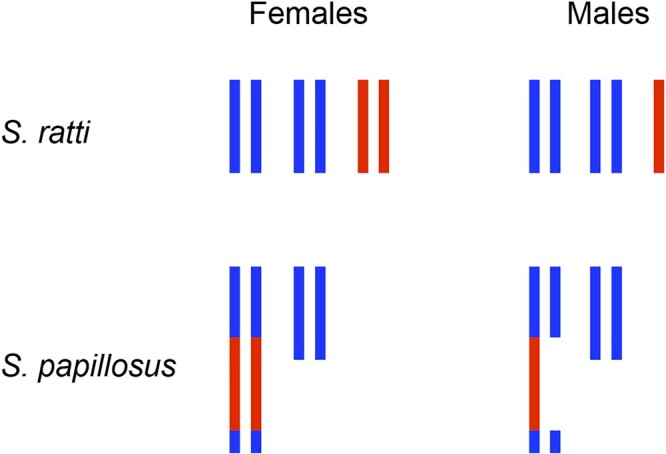
DNA elimination in *Strongyloides* spp. Chromosomal configuration in females (left) and males (right) in *S. ratti* (top) and *S. papillosus* (bottom). Chromosomes and chromosomal regions present in 2 copies in both sexes are in blue; chromosomes and regions present in 2 copies in females but only 1 copy in males are in red. Note in *S. ratti*, 1 whole chromosome is lost in males, while in *S. papillosus*, only part of a chromosome is lost during programmed DNA elimination [from Streit *et al.* (2016)]

DNA elimination is also known to occur in another group of nematodes, the genus *Strongyloides* [reviewed in (Streit *et al.* 2016) and (Albertson *et al.* 1979; Nemetschke *et al.* 2010; Hunt *et al.* 2016)]. DNA elimination in these nematodes functions in environmentally influenced sex determination and occurs in 2 forms: (1) elimination of an entire chromosome or (2) loss of only a portion of a chromosome (Fig. 12). In parthenogenetically produced males of the rat parasite *S.* *ratti*, only 1 of the 2 X chromosomes from the mother is inherited (2*n* = 6 in females and 2*n* = 5 in males; Nigon and Roman 1952; Harvey and Viney 2001). Loss of the noninherited X chromosome likely occurs during mitotic oocyte maturation. The mechanism for chromosome loss and how it is induced by the host immune status is unknown. In another *Strongyloides*, *S*trongyloides *papillosus*, the X chromosome appears to have been inserted into one of the autosomes. In males, this inserted X is randomly eliminated in 1 of the 2 homologous chromosomes. This suggests that chromosome breaks occur in one of the chromosomes and the X region is not segregated. The mechanism for how this occurs is unknown, but could be similar to the process in *Ascaris* described above (breaks and differential localization of CENP-A/kinetochores). Overall, DNA elimination in the *Strongyloides* differs from that observed in ascarids and *Oscheius* as follows (Streit *et al.* 2016): (1) DNA loss is involved in sex determination and not soma vs germline differentiation, (2) DNA elimination leads to reduction in half of the gene dosage compared to complete gene loss, (3) the majority of DNA lost is not repetitive DNA, (4) DNA elimination is facultatively induced by environmental cues to produce males in *Strongyloides*, whereas it always occurs as a normal part of somatic development of ascarids, and (5) DNA elimination occurs in multiple early embryo cells in ascarids. The differences and large phylogenetic distance between these nematode groups could suggest that DNA elimination may have evolved independently in these groups.

### Perspectives

The great diversity and importance of nematodes have led to substantial work on their biology, ecology, and genomes, creating important insights and tools that will be relevant to broadly understanding the function, organization, and malleability of nematode chromosomes. Additional and unique insights into the evolution of chromosomes will benefit from the combination of genome sequence analysis, modifications of genomes, and tests of hypotheses in experimentally tractable nematodes. Initially, it may be most feasible to perform such tests in *C. elegans* including wild isolates that are substantially diverged in many respects, and in more distantly related nematodes in Clade V. These efforts in more closely related nematodes may help to usher in a new era of chromosome biology where systems that have evolved to uniquely serve specific nematode species or clades are inactivated and replaced by mechanistically distinct biological counterparts from other species. For example, analysis of nematode chromosomes has provided particular insights into holocentric chromosomes and their impact on meiosis. If monocentric nematodes can be identified, then this might provide an opportunity to study the transition to holocentricity, which likely happened early on in nematode evolution. The contributions of holocentricity to centromeric organization, genome architecture, DNA repair pathways and chromosome organization and function should provide insights into nematode chromosome evolution. Similarly, studies of transitions between modes of genome silencing could illuminate the mechanisms by which DNA methylation, small RNA, and histone silencing pathways are lost, created, or adapted.

Analysis of chromosomes in nematodes over the past almost 150 years has identified only a few nematodes that undergo DNA elimination. Notably, DNA elimination is now known to occur in Clade V nematodes including *Oscheius* species and *Caenorhabditis monodelphis*. If additional nematodes that undergo DNA elimination are identified by comprehensive genome sequence analysis of germline and somatic tissues, this may help to address key questions concerning the selective pressures that allow this process to evolve. Why is this mechanism of gene silencing used so rarely? Does DNA elimination more broadly serve to remodel chromosome ends and if so, why? Development of a variety of additional tools and methods for gene and chromosome manipulation in DNA eliminating nematodes will be required to address for example whether this process is essential for somatic development and sex determination. Manipulation of genes with potential relevance to chromosome biology, manipulation of genome sequences, and small-scale evolution experiments may distill important facets relevant to genome plasticity, such as regulation of synteny, which appears to be strongly conserved at the level of whole chromosomes. As nearly complete haploid genomes are becoming easier to assemble, their analysis may set the stage for insightful experiments that comprehensively address synteny, chromosome organization, and gene and repeat content, which may shed new light on a long-standing question in biology: how do chromosomes and genomes evolve?
